# Rheumatoid arthritis and cardiovascular disease associations in the UK Biobank

**DOI:** 10.1186/s12916-025-04431-1

**Published:** 2025-11-03

**Authors:** Janek Salatzki, Dorina-Gabriela Condurache, Stefania D’Angelo, Ahmed M. Salih, Liliana Szabo, Adil Mahmood, Elizabeth M. Curtis, Steffen E. Petersen, Andre Altmann, Norbert Frey, Florian André, Nicholas C. Harvey, Zahra Raisi-Estabragh

**Affiliations:** 1 William Harvey Research Institute, NIHR Barts Biomedical Research Centre, Queen Mary University of London, Charterhouse Square, London, EC1M 6BQ UK; 2 Department of Cardiology, Angiology and Pneumology, Heidelberg University Hospital, 69120 Heidelberg, Germany; 3 Barts Heart Centre, St Bartholomew’s Hospital, Barts Health NHS Trust, West Smithfield, London, EC1A 7BE UK; 4 MRC Lifecourse Epidemiology Centre, University of Southampton, Southampton, SO16 6YD UK; 5 Department of Population Health Sciences, University of Leicester, Leicester, UK; 6 PRIME Lab, Scientific Research Center, University of Zakho, Zakho, Kurdistan Region Iraq; 7 Heart and Vascular Center, Semmelweis University, Budapest, Hungary; 8 NIHR Southampton Biomedical Research Centre, University of Southampton and University Hospital Southampton NHS Foundation Trust, Southampton, UK; 9 Department of Medical Physics and Biomedical Engineering, The UCL Hawkes Institute, University College London, London, UK

**Keywords:** Rheumatoid arthritis, Cardiovascular disease, Ischaemic heart disease, Pericardial disease, Epidemiology, Risk factors, Mendelian randomisation, Causal association, UK Biobank

## Abstract

**Background:**

This study evaluated observational and causal relationships between rheumatoid arthritis (RA) and cardiovascular disease and imaging phenotypes in the UK Biobank.

**Methods:**

RA was defined using linked hospital records, self-reported diagnostics, and medication data. Controls were participants without a record of RA. Cardiovascular diseases (CVDs) were defined using linked hospital records over an average of 14 years of prospective follow-up, including: ischaemic heart diseases (IHD), acute myocardial infarction (AMI), atrial fibrillation, any arrhythmia, non-ischaemic cardiomyopathies, pericardial disease, stroke, peripheral vascular disease, and venous thromboembolism. For participants with cardiovascular magnetic resonance (CMR) available as part of the UK Biobank Imaging Study, we considered measures of cardiac structure and function extracted using automated pipelines. Associations of RA with prevalent and incident CVDs were calculated using logistic and Cox regression. Linear regression was used to examine associations with CMR metrics. Models were adjusted for demographic, lifestyle, and clinical confounders. Causal associations were assessed using two-sample Mendelian randomisation. Genetic instruments for RA (22,350 cases and 74,823 controls), nine CVDs (FinnGen, *n* = 224,737), and 11 CMR phenotypes (UK Biobank) were extracted and associations assessed using inverse-variance weighting with pleiotropy adjustments and multiple testing corrections.

**Results:**

The analysis included 1,436 RA cases (mean age 59.9 years; 70.6% female) and 476,975 controls (mean age 56.5 years; 54.3% female). Participants with RA lived in more socioeconomically deprived areas (as per the Townsend Deprivation Index), had lower physical activity levels, were more likely to smoke, and had a higher baseline prevalence of CVDs. In fully adjusted models, participants with RA had a significantly higher hazard of multiple incident CVDs, with the greatest risks related to pericardial disease (HR 2.63 (1.85, 3.74)), heart failure (HR 1.68 (1.42, 1.99)), and AMI (HR 1.53 (1.20, 1.96)). Mendelian Randomisation analyses supported causal links between RA and AMI (OR 1.07 (1.02, 1.09), *p* = 0.009), arrhythmias (OR 1.05 (1.02, 1.06), *p* = 0.0007), and IHD (OR 1.05 (1.01, 1.06), *p* = 0.036). No significant associations were identified between RA and CMR phenotypes.

**Conclusions:**

People with RA have a heightened risk of multiple prevalent and incident CVDs, independent of shared risk factors, with suggestions of causal links with IHD, AMI, and arrhythmias.

**Supplementary Information:**

The online version contains supplementary material available at 10.1186/s12916-025-04431-1.

## Background

Rheumatoid arthritis (RA) is a chronic systemic inflammatory autoimmune disorder. While the condition primarily targets the synovial joints, multiple organ systems are commonly affected [1]. 

Cardiovascular manifestations of RA are major determinants of prognosis and quality of life [2, 3]. Cardiovascular disease (CVD) is the leading cause of death in patients with RA, who have a 50% higher risk of cardiovascular mortality compared to the general population [3]. This increased burden results from a complex interplay of systemic inflammation, immune dysregulation, traditional cardiovascular risk factors and adverse effects of RA-specific therapies [4–6]. However, the precise biological mechanisms linking RA to increased CVD risk remain poorly understood.

Previous research has examined cardiovascular manifestations in small, select clinical cohorts of RA patients [7–10], but these studies are limited by short follow-up and low statistical power. As a result, their findings may not generalise to the broader population of RA patients with varying levels of disease activity.

Cardiovascular magnetic resonance (CMR) offers a valuable tool for detecting early and subclinical cardiovascular changes in the context of RA. However, prior studies have largely focused on specific cardiac parameters in limited populations [11–13]. The clinical utility of CMR in unselected RA patients remains unclear.

Traditional observational analyses, while informative, are limited by residual confounding and reverse causation, making it challenging to definitively assess the causality of reported associations. Mendelian Randomisation (MR) is a statistical method that can strengthen causal inference by leveraging genetic variants as instrumental variables (IV), which are randomly allocated at conception and thus less susceptible to confounding and reverse causation [14].

Previous studies have examined the associations of RA with individual clinical CVD outcomes such as coronary artery disease (CAD) [15], ischaemic heart disease (IHD) and acute myocardial infarction (AMI) [16], heart failure (HF) [17, 18], atrial fibrillation (AF) [19, 20], and stroke [21]. However, results have been inconsistent, and few have assessed a broad range of CVD outcomes within a unified framework.

To address these gaps, we present a comprehensive study that integrates observational epidemiology, MR, and CMR imaging within a single analytic framework. This triangulated approach enhances causal inference and offers a multidimensional assessment of cardiovascular risk in RA. By examining a broad spectrum of CVD outcomes—including under-recognised conditions—and incorporating imaging data, our study provides a more holistic understanding of RA-related cardiovascular involvement than previously available. The findings could guide early cardiovascular screening, inform risk factor management, and support integrated care for RA patients.

The UK Biobank is a very large multimodal biomedical research resource, offering detailed participant characterisation, genotyping, and prospective tracking of incident health outcomes through linkage to electronic health records [22]. The UK Biobank Imaging Study further enhances this potential by enabling the evaluation of cardiac structure and function using CMR in a large subset of participants [23].

Specifically, we aim to (1) explore the prevalence and incidence of CVDs and associated cardiovascular risk factors in RA participants compared to non-RA controls; (2) investigate the causal relationships between RA and CVD outcomes using MR analyses, leveraging data from large publicly available genomic datasets; and (3) examine the links between RA and CMR measures of cardiac structure and function, using both observational analyses and MR approaches.

## Methods

### Study design

**Fig. 1:**
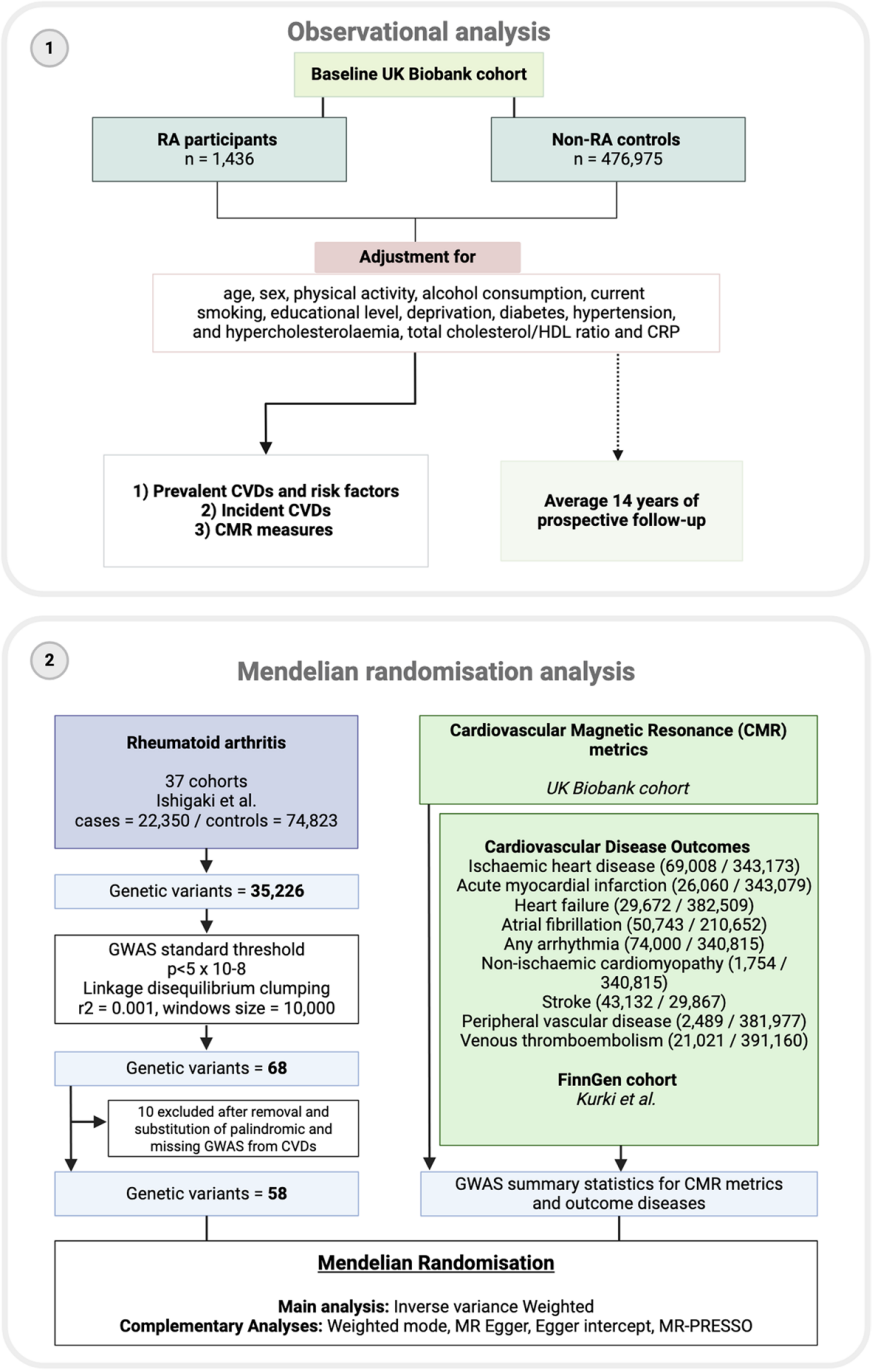
Overview of the present study design. Footnote Fig. 1: (1) We performed an observational analysis using UK Biobank data, a cohort of over 500,000 individuals with baseline assessments and linkage to national health records for incident cardiovascular events. Rheumatoid arthritis (RA) participants were defined using diagnostic records and RA-specific medication use, while controls were participants without any RA diagnosis during follow-up (average 14 years). Cardiovascular disease (CVD) outcomes were identified via ICD codes. In a subset of participants, cardiovascular magnetic resonance (CMR) imaging data were analysed to assess cardiac structure and function. Key covariates, including demographic and lifestyle factors, were adjusted for in the analysis. (2) We initially identified 35,226 genetic variants, which were filtered using a GWAS standard threshold (*p* < 5 × 10^−8^) and linkage disequilibrium clumping (*r*.^2^ = 0.001, window size = 10,000), resulting in 68 variants. After excluding 10 variants due to removal and substitution of palindromic or missing GWAS data from CVDs, 58 variants remained. Mendelian randomisation was performed using inverse variance weighted method, with complementary analysis (weighted median, weighted mode, MR Egger, Egger intercept, MR-PRESSO) and multiple testing correction (Bonferroni method)

We explored the observational associations between RA and the risk of incident and prevalent CVDs in the UK Biobank. To assess the causality of these associations, we performed MR analyses using data from large-scale genome-wide association studies (GWASs). Details of the study design and analyses can be found in Fig. 1.

### Observational analysis

#### Study population

The UK Biobank is a large population-based cohort of over half a million individuals recruited between 2006 and 2010 from across the United Kingdom [22]. Baseline assessments included touchscreen questionnaires, nurse-led interviews, physical measurements and blood sampling. The UK Biobank Imaging Study commenced in 2014 and includes detailed CMR imaging for a large subset of the original cohort [23]. Linkage to national health databases, such as Hospital Episode Statistics (HES) and Office for National Statistics (ONS) mortality data, enables prospective monitoring of incident health events for the entire cohort.

### Ascertainment of exposure

RA status at baseline recruitment was defined using a combination of diagnostic records and the use of relevant medications. RA diagnosis was identified from self-reported data during nurse-led interviews at baseline assessment or linked HES records using International Classification of Disease (ICD) codes (detailed in Additional File 1: Table 1). Records of specific medications for RA were identified from self-reported data. Any formulation of medications falling within the following classes was included: systemic steroids, conventional disease-modifying anti-rheumatic drugs (DMARDs), and novel biological agents (full list in Additional File 1: Table 2). Participants with a record of RA diagnosis but no corresponding record of RA-specific medications were excluded. This approach was used to ensure accurate labelling of RA status, minimising the risk of misclassification, as previously described [24]. The control group consisted of UK Biobank participants with no record of RA or relevant medication during the available follow-up period.

**Table 1:** Baseline characteristics of the study population

	**Whole set**	**No RA**	**RA**
*N* (%)	478,411	476,975 (99.7)	1436 (0.3)
Age, mean (SD)	56.6 (8.1)	56.5 (8.1)	59.9 (6.9)
Sex, *N* (%)			
Female	259,837 (54.3)	258,823 (54.3)	1014 (70.6)
Male	218,574 (48.7)	218,152 (45.7)	422 (29.4)
Index of Multiple Deprivation	12.8 (7.4,22.9)	12.8 (7.4,22.9)	14.1 (8.4,26.6)
Educational level, *N* (%)			
College/University	153,899 (32.2)	153,611 (32.3)	288 (20.1)
Other professional qualification/A levels	155,757 (32.6)	155,335 (32.6)	422 (29.4)
GCSE or less	160,451 (33.5)	159,764 (33.5)	687 (47.8)
Missing	8304 (1.7)	8265 (1.7)	39 (2.7)
Physical activity (IPAQ score MET min/week)	1794 (820,3573)	1794 (822,3573)	1039.5 (346.5,2599.5)
Body mass index (kg/m^2^), mean (SD)	27 (4.8)	27 (4.8)	28 (5.6)
Alcohol intake frequencies, *N* (%)			
Never	34,115 (7.1)	33,859 (7.1)	256 (17.8)
Special occasions only	53,192 (11.1)	52,951 (11.1)	241 (16.8)
1–3 times a month	53,378 (11.2)	53,199 (11.2)	179 (12.5)
Once or twice a week	124,912 (26.1)	124,529 (26.1)	383 (26.7)
3–4 times a week	112,719 (23.6)	112,527 (23.4)	192 (13.4)
Daily or almost daily	99,583 (20.8)	99,399 (20.8)	184 (12.8)
Missing	512 (0.1)	511 (0.1)	1 (0.1)
Current smoking status, *N* (%)			
Never	258,449 (54.0)	257,856 (54.1)	593 (41.3)
Previous	167,798 (35.1)	167,129 (35.0)	669 (46.6)
Current	50,315 (10.5)	50,150 (10.5)	165 (11.5)
Missing	1849 (0.4)	1840 (0.4)	9 (0.6)
Medication for rheumatoid arthritis			
Steroids	-	-	9 (0.6)
Conventional DMARD	-	-	1371 (95.5)
Biologic DMARD	-	-	56 (3.9)
Diabetes, *N* (%)	27,362 (5.7)	27,211 (5.7)	151 (10.5)
Hypertension, *N* (%)	139,110 (29.1)	138,408 (29.0)	702 (48.9)
High cholesterol, *N* (%)	88,496 (18.5)	88,060 (18.5)	436 (30.4)
**Prevalent CVDs**			
Ischaemic heart disease, *N* (%)	22,986 (4.8)	22,820 (4.8)	166 (11.6)
Myocardial infarction, *N* (%)	11,273 (2.4)	11,189 (2.4)	84 (5.9)
Heart failure, *N* (%)	2218 (0.5)	2200 (0.5)	18 (1.3)
Atrial fibrillation, *N* (%)	2536 (0.5)	2513 (0.5)	23 (1.6)
Any arrhythmia, *N* (%)	5067 (1.1)	5031 (1.1)	36 (2.5)
Non-ischaemic cardiomyopathy, *N* (%)	620 (0.1)	616 (0.1)	4 (0.3)
Pericardial disease, *N* (%)	652 (0.1)	640 (0.1)	12 (0.8)
Stroke, *N* (%)	7929 (1.7)	7879 (1.7)	50 (3.5)
Peripheral vascular disease, *N* (%)	1716 (0.4)	1700 (0.4)	16 (1.1)
Venous thromboembolism, *N* (%)	6405 (1.3)	6334 (1.3)	71 (4.9)
**Incident CVDs**			
Ischaemic heart disease, *N* (%)	35,458 (7.4)	35,272 (7.4)	186 (13.0)
Acute myocardial infarction, *N* (%)	11,212 (2.3)	11,148 (2.3)	64 (4.5)
Heart failure, *N* (%)	16,547 (3.5)	16,407 (3.4)	140 (9.8)
Atrial fibrillation, *N* (%)	29,043 (6.1)	28,895 (6.1)	148 (10.3)
Any arrhythmia, *N* (%)	25,370 (5.3)	25,231 (5.3)	139 (9.7)
Non-ischaemic cardiomyopathy, *N* (%)	2795 (0.6)	2778 (0.6)	17 (1.2)
Pericardial disease, *N* (%)	2991 (0.6)	2959 (0.6)	32 (2.2)
Stroke, *N* (%)	10,937 (2.3)	10,878 (2.3)	59 (4.1)
Peripheral vascular disease, *N* (%)	7747 (1.6)	7689 (1.6)	58 (4.0)
Venous thromboembolism, *N* (%)	8730 (1.8)	8682 (1.8)	48 (3.3)

Discrete variables are expressed as number (%). Continuous variables are expressed as mean (SD) or median (25th percentile, 75th percentile) depending on distribution

*CVD* cardiovascular diseases, *DMARD* disease-modifying anti-rheumatic drug, *GCSE* General Certificate of Secondary Education, *IPAQ* International Physical Activity Questionnaire, *MET* Metabolic Equivalent of Task, *RA* rheumatoid arthritis

**Table 2:** Association of rheumatoid arthritis with prevalent cardiovascular diseases and vascular risk factors

**Outcome**	**Model 1**	**Model 2 (MICE)**
	*OR (95% CI), p-value*	
Ischaemic heart disease	2.03 (1.72,2.40) < 0.001	1.59 (1.32,1.92) < 0.001
Acute myocardial infarction	2.03 (1.62,2.54) < 0.001	1.76 (1.28,2.26) < 0.001
Heart failure	2.14 (1.34,3.42) 0.001	1.42 (0.88,2.29) 0.15
Atrial fibrillation	2.34 (1.55,3.55) < 0.001	2.00 (1.31,3.04) 0.001
Any arrhythmia	2.01 (1.44,2.80) < 0.001	1.61 (1.15,2.25) 0.006
Non-ischaemic cardiomyopathy	1.84 (0.69,4.93) 0.24	1.38 (0.51,3.73) 0.52
Pericardial disease	5.56 (3.13,9.88) < 0.001	4.62 (2.57,8.27) < 0.001
Stroke	1.76 (1.33,2.34) < 0.001	1.17 (0.88,1.57) 0.29
Peripheral vascular disease	2.48 (1.51,4.07) < 0.001	1.47 (0.88,2.45) 0.14
Venous thromboembolism	3.24 (2.54,4.12) < 0.001	1.96 (1.53,2.51) < 0.001
Diabetes	1.67 (1.41,1.98) < 0.001	0.99 (0.82,1.19) 0.89
Hypertension	1.95 (1.75,2.17) < 0.001	1.53 (1.36,1.73) < 0.001
Hypercholesterolaemia	1.50 (1.34,1.69) < 0.001	1.15 (1.00,1.32) 0.05

Results are reported as ORs with 95% CI and *p*-values for each CVD outcome and vascular risk factors, across two models. Model 1 was adjusted for age and sex. Model 2 incorporated additional adjustments for physical activity, alcohol consumption, smoking status, educational level, deprivation, diabetes, hypertension, hypercholesterolaemia, total cholesterol/HDL ratio and CRP. MICE was used to estimate missing values, ensuring a more complete dataset and avoiding bias, with results combined using Rubin’s rules to maintain accuracy. *CI* confidence interval, *OR* odds ratio, *MICE* multiple imputation by chained equations

### Ascertainment of cardiovascular outcomes

The baseline status of key metabolic morbidities, including diabetes, hypertension, and hypercholesterolaemia, was determined using an algorithm-based approach that integrated self-reported data during nurse-led interviews and ICD codes from linked HES records. Further details are provided in Additional File 1: Table 3. The following prevalent and incident CVDs were included: IHD, AMI, HF, AF, arrhythmias, non-ischaemic cardiomyopathy (NICM), pericardial disease, stroke, peripheral vascular disease (PVD) and venous thromboembolism (VTE). The index date was the date of recruitment into the UK Biobank. Prevalent events were conditions present at baseline. Incident events were those occurring for the first time after the baseline assessment. All cardiovascular conditions were defined based on ICD codes from linked hospital records using the latest available censor date of 31 October 2022.

**Table 3:** Association of rheumatoid arthritis with incident cardiovascular diseases

**Outcome**	**Model 1**	**Model 2 (MICE)**
	*HR (95% CI), p-value*	
Ischaemic heart disease	1.72 (1.49,1.98) < 0.001	1.38 (1.19,1.60) < 0.001
Acute myocardial infarction	1.95 (1.53,2.50) < 0.001	1.53 (1.20,1.96) 0.001
Heart failure	2.54 (2.15,3.00) < 0.001	1.68 (1.42,1.99) < 0.001
Atrial fibrillation	1.50 (1.27,1.76) < 0.001	1.24 (1.05,1.46) 0.01
Any arrhythmia	1.66 (1.40,1.96) < 0.001	1.36 (1.15,1.61) < 0.001
Non-ischaemic cardiomyopathy	1.92 (1.19,3.10) 0.007	1.48 (0.92,2.39) 0.11
Pericardial disease	3.37 (2.37,4.77) < 0.001	2.63 (1.85,3.74) < 0.001
Stroke	1.56 (1.21,2.02) 0.001	1.30 (1.00,1.68) 0.05
Peripheral vascular disease	2.26 (1.75,2.93) < 0.001	1.43 (1.11,1.89) 0.006
Venous thromboembolism	1.64 (1.24,2.18) 0.001	1.33 (1.00,1.76) 0.05

Results are reported as HR with 95% CI and *p*-values for each CVD outcome and vascular risk factors, across two models. Model 1 was adjusted for age and sex. Model 2 is adjusted for ethnicity, physical activity, alcohol consumption, smoking status, educational level, deprivation, diabetes, hypertension, hypercholesterolaemia, total cholesterol/HDL ratio and CRP

Model 3, using multiple imputation (MICE) for missing data (35% of the sample), included the same covariates as Model 2, with the addition of diabetes, hypertension, and hypercholesterolaemia. *CI* confidence interval, *HR* hazard ratio, *MICE* multiple imputation by chained equations

### Ascertainment of covariates

Covariates were identified from UK Biobank baseline assessment, including age, sex, physical activity, smoking status, alcohol use, education and socio-economic status (based on the Townsend Deprivation Index). We additionally included key cardiometabolic risk factors: diabetes, hypertension, hypercholesterolaemia, total cholesterol to HDL cholesterol ratio, and C-reactive protein (CRP) to account for their potential confounding influence on cardiovascular outcomes. Ethnicity was not included as a covariate, as the analysis was restricted to participants of White/European ancestry to minimise population stratification bias.

### CMR image acquisition and analysis

CMR examinations were performed on 1.5 Tesla scanners (MAGNETOM Aera, Syngo Platform VD13A, Siemens Healthcare, Erlangen, Germany) in dedicated imaging units in accordance with predefined protocols [25]. Images were processed and analysed using automated pipelines [26]. The following CMR phenotypes were considered: left ventricular (LV) wall thickness (WT), LV mass (LVM), LV end-diastolic volume (LVEDV), LVM to LVEDV ratio, LV stroke volume (LVSV), LV ejection fraction (LVEF), LV global functional index (LVGFI), LV global longitudinal strain (LV GLS), right ventricle end-diastolic volume (RVEDV), right ventricle stroke volume (RVSV), right ventricle ejection fraction (RVEF), aortic distensibility (AoD) (Additional File 1: Table 4).

### Mendelian randomisation analysis

To investigate the causal relationship between RA and cardiovascular outcomes, we employed a two-sample MR method. We reviewed existing literature to identify GWASs relevant to RA (as the exposure), nine CVDs, and eleven CMR metrics (as the outcomes). From the identified GWAS studies, we selected appropriate genetic variants (single-nucleotide polymorphisms, SNPs) as IVs to explore these causal relationships. The two-sample MR framework outlining the study design is summarised in Fig. 1

### Data sources

#### Exposure

##### Rheumatoid arthritis

Summary statistics data for RA were obtained from a meta-analysis of GWASs detailed by Ishigaki et al. [27]. This study included 276,020 samples from five ancestral groups: European, East Asian, African/African American, South Asian, and Arab. For this analysis, only data from the European population were used, encompassing 22,350 cases and 74,823 controls. All RA cases in this GWAS were defined based on the 1987 American College of Rheumatology (ACR) criteria [28] or the 2010 ACR/European League Against Rheumatism [29] criteria or were diagnosed with RA by a professional rheumatologist.

#### Outcomes

##### Cardiovascular diseases

The outcome dataset for CVDs was derived from the FinnGen GWAS database (https://www.finngen.fi/en), which included 224,737 participants from Finnish ancestry [30]. The FinnGen consortium is an ongoing project launched in Finland in 2017, collecting genetic and electronic health record information, aiming to explore the human genome. Nine CVDs were used as outcomes in this study. These included IHD, AMI, HF, AF, any arrhythmia, NICM, stroke, PVD, and VTE. Detailed descriptions of sample size, case proportion, and specific population characteristics are presented in Additional File 1: Table 5.

##### Cardiovascular magnetic resonance (CMR) metrics

The outcome dataset for CMR metrics was obtained from six GWAS studies using data from the UK Biobank. Aung et al. (2022 June) [31] provided measurements of the right ventricle (RVESV, RVEDV and RVEF) from a sample of 29,506 subjects. Aung et al. (2019 Oct) [32] contributed data on LVM from 16,923 subjects. Genetic data on LVSV, LVEDV, LVESV and LVEF was available from Pirruccello et al. [33] on 40,000 individuals. Nauffal et al. [34] provided data on native myocardial T1 times with a sample size of 41,505. Furthermore, Fung et al. [35] provided data on arterial stiffness index (ASI) from 127,121 individuals, and Francis et al. [36] supplied data on AoD from 32,590 individuals (Additional File 1: Table 6).

##### Genetic instrument selection

A screening protocol for SNPs was applied, including (1) extraction of genetic variants that passed the standard GWAS *p-*value threshold for genome-wide significance (*p* < 5 × 10^−8^); (2) application of linkage disequilibrium (LD) clumping (*r*^2^ = 0.001 within a 10,000 kb window) to ascertain independent SNPs, using the R package “ieugwasr” [37]; (3) selection of proxy SNPs based on LD if the selected SNPs were palindromic or missing from the CVD GWASs. To qualify as a proxy, we used the following criteria: European population, *r*^2^ > 0.1, and base pair windows of 10,000 bases; (4) verification that proxy SNPs remained significantly associated with RA based on the GWAS summary statistics, with non-qualifying proxies being removed (Additional File 1: Table 7).

### Additional Mendelian randomisation analysis in East Asian populations

To assess the generalisability of our findings across ethnic groups, we conducted an additional MR analysis using summary-level data from an East Asian GWAS of RA [27]. A total of 50 independent SNPs were retained after exclusion of palindromic and poorly matched proxies. These SNPs were used to evaluate causal associations with cardiovascular outcomes in publicly available East Asian GWAS datasets (The BioBank Japan Project https://pheweb.jp/) [38]. The outcomes considered included AF, HF, IHD, AMI, dilated and hypertrophic cardiomyopathy, peripheral artery disease, and stroke. Additional File 1: Table 8 provides comprehensive information on cohort sizes, case frequencies, and phenotype definitions across the East Asian datasets.

### Statistical analyses

#### Observational analysis

Statistical analysis was performed using STATA software version 17 (StataCorp LLC, College Station, TX, USA). Discrete variables were expressed as numbers and percentages, and continuous variables as mean (SD) or median (25th, 75th percentiles). Logistic regression models assessed the associations between RA and prevalent CVDs (odds ratios, ORs), while Cox proportional hazards models were used for incident CVDs (hazard ratios, HRs). For associations with CMR metrics, linear regression models (beta coefficients) were used.

We created two models with different layers of adjustment: Model 1 (age, sex) and Model 2 (age, sex, physical activity, alcohol consumption, current smoking, educational level, deprivation, diabetes, hypertension, hypercholesterolaemia, total cholesterol/HDL ratio and CRP). Missing covariate data in the fully adjusted models (35% of the sample) were imputed using multiple imputation by chained equations (MICE), and results were combined using Rubin’s rules to account for the variability introduced by the imputation process [39]. Results are also presented for complete case analysis as sensitivity checks alongside the imputed models.

### Mendelian randomisation

The causal relationships were assessed using the inverse variance weighted (IVW) method. To enhance this approach, several robust MR methods were used in the sensitivity analysis, including weighted median, weighted mode and MR Egger methods [40]. The Egger intercept method was used to assess the pleiotropic effect of the selected IVs, and the intercept term *p* < 0.05 indicated significant directional pleiotropy. Additionally, MR Pleiotropy Residual Sum and Outlier (MR-PRESSO) was employed to detect possible outliers and investigate horizontal pleiotropy, indicated by a global *p* < 0.05 [41].

Power calculations were conducted to determine whether our study was adequately powered to detect true causal effects between RA and CVD outcomes. We assessed the association of each SNP with the risk of CVD, applying the Bonferroni correction, with statistical significance set at α < 0.0056 (0.05/9). The power of the MR estimates was calculated using the online tool mRnd.

(https://shiny.cnsgenomics.com/mRnd/) [42] (Additional File 1: Table 9). For the East Asian MR analysis, power calculations were also performed, and a Bonferroni correction was applied to account for multiple testing, setting the threshold for statistical significance at α < 0.00625 (0.05/8).

## Results

### Observational analysis of RA and cardiovascular outcomes

#### Baseline characteristics

We identified 1,436 participants with RA and 476,975 control participants with no record of RA at any time (Table 1). At baseline recruitment, most RA patients (95.5%) were using conventional DMARDs, a smaller group (3.9%) received biologic agents, and 0.6% were on systemic steroids. Compared with controls, participants with RA were older (mean age 59.9 vs. 56.5 years), predominantly female (70.6% vs. 54.3%), more sedentary (1040 vs 1794 MET min/week), and had a higher BMI (28 vs. 27 kg/m^2^). The RA group had lower educational attainment and were more likely to be former or current smokers (58.1% vs 45,5%).

#### Prevalent CVDs and VRFs

Participants with RA had a significantly higher burden of metabolic morbidities compared to controls (Table 1), with more than 10% of participants having a record of diabetes (vs. 5.7%), nearly half having hypertension (vs. 29.0%), and 30.4% having hypercholesterolaemia (vs. 18.5%). The prevalence of all CVDs considered was higher in the RA group compared to controls (Table 1). AMI was the most common, occurring at more than twice the rate of that in controls (5.9% vs. 2.4%), followed by VTE (4.9% vs. 1.3%) and stroke (3.5% vs. 1.7%).

**Fig. 2:**
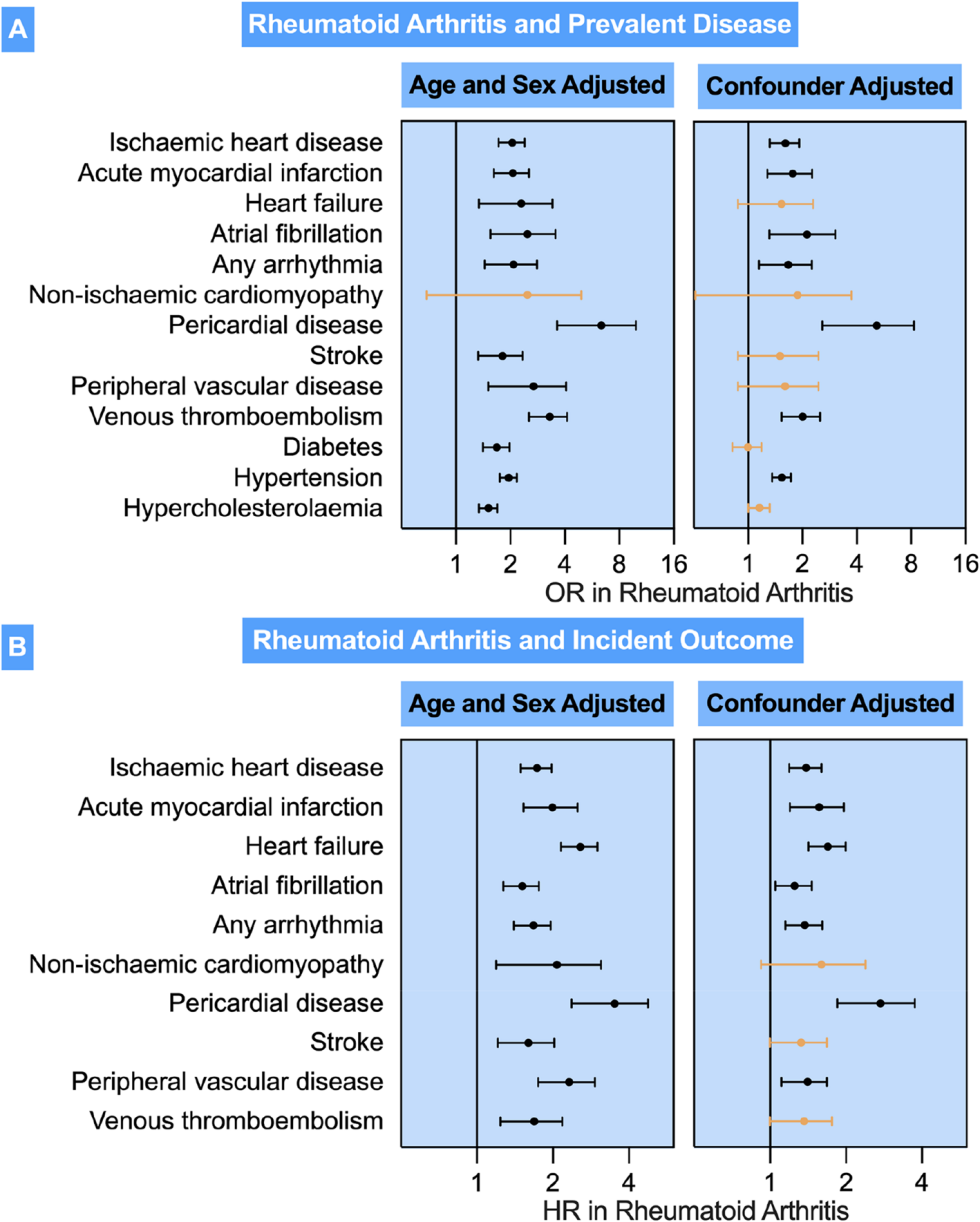
Association of rheumatoid arthritis with prevalent diseases and incident outcomes. Footnote Fig. 2: **A** Results are odds ratios (OR) from logistic regression models. **B** Results are hazard ratios (HR) from Cox proportional hazards models. The diseases listed are set as the model outcome and rheumatoid arthritis is the exposure of interest. The “age and sex adjusted model” are adjusted for age and sex. The “confounder adjusted” models are adjusted for age, sex, physical activity, alcohol consumption, current smoking, educational level, and deprivation (Index of Multiple Deprivation), as well as diabetes, hypertension, hypercholesterolaemia, total cholesterol/HDL ratio and CRP. Missing values were assumed to be missing at random and multiple imputation by chained equations (MICE) was used to impute missing data (Model 3 MICE). Each bar corresponds to a separate model. The point and bars indicate the point estimate and 95% CI, respectively. The orange bars indicate statically nonsignificant associations

In fully adjusted models, the strongest associations were between RA and higher odds of pericardial disease (OR 4.62, 95% CI: 2.57–8.27, *p* < 0.001), AF (OR 2.00, 95% CI: 1.31–3.04, p = 0.001) and VTE (OR 1.96, 95% CI: 1.53–2.51, *p* < 0.001). Further significant associations were observed between RA and AMI (OR 1.76, 95% CI: 1.28–2.29, *p* < 0.001) and hypertension (OR 1.53, 95% CI: 1.36–1.73, *p* < 0.001). Associations with HF, NICM, stroke, diabetes and hypercholesterolaemia were not statistically significant after adjusting for demographic, lifestyle, and baseline metabolic morbidities (Table 2, Fig. 2). The results of models with imputed covariates were consistent with those from complete case analyses (Additional File 1: Table 10).

#### Incident CVDs

During an average follow-up of 14 years, participants with RA showed a higher incidence of all CVDs considered compared to controls (Table 1). The three most common incident CVDs in the RA group were AF (10.3% vs. 6.1%), heart failure (9.8% vs. 3.4%), and AMI (4.5% vs. 2.3%).

In multivariable Cox regression models adjusted for age and sex (Model 1), RA was significantly associated with increased risks of all incident CVD (Table 3, Fig. 2). These associations remained robust after full adjustment for additional confounding factors (Model 2), except for NICM, stroke and VTE. The strongest associations were observed for pericardial diseases (HR 2.63, 95% CI: 1.85–3.74, *p* < 0.001), HF (HR 1.63, 95% CI: 1.42–1.99, *p* < 0.001), and AMI (HR 1.53, 95% CI: 1.20–1.96, *p* = 0.001) (Table 3). These were consistent with results in complete-case analyses (Additional File 1: Table 11).

### Mendelian randomisation analysis of the RA-cardiovascular outcome association

#### SNP selection and validation

Based on the power calculation results, the analyses were adequately powered to detect true associations between RA and the nine CVDs. The causal effects detectable with 90% statistical power are detailed in Additional File 1: Table 9. We extracted 68 independent SNPs based on genome-wide significance from the RA GWAS. After applying LD clumping, 11 SNPs were not available in the CVD GWAS datasets and nine were palindromic, requiring substitution with their proxies. One SNP was excluded due to the lack of an appropriate proxy (*R*^2^ < 0.1). Six proxy candidates were removed as they were not significantly associated with RA. Consequently, 61 SNPs remained, with three additional exclusions due to persistent palindromic issues, resulting in a final list of 58 SNPs used for the MR analysis (Additional File 1: Table 7).

#### Causal association between RA and CVDs

**Fig. 3:**
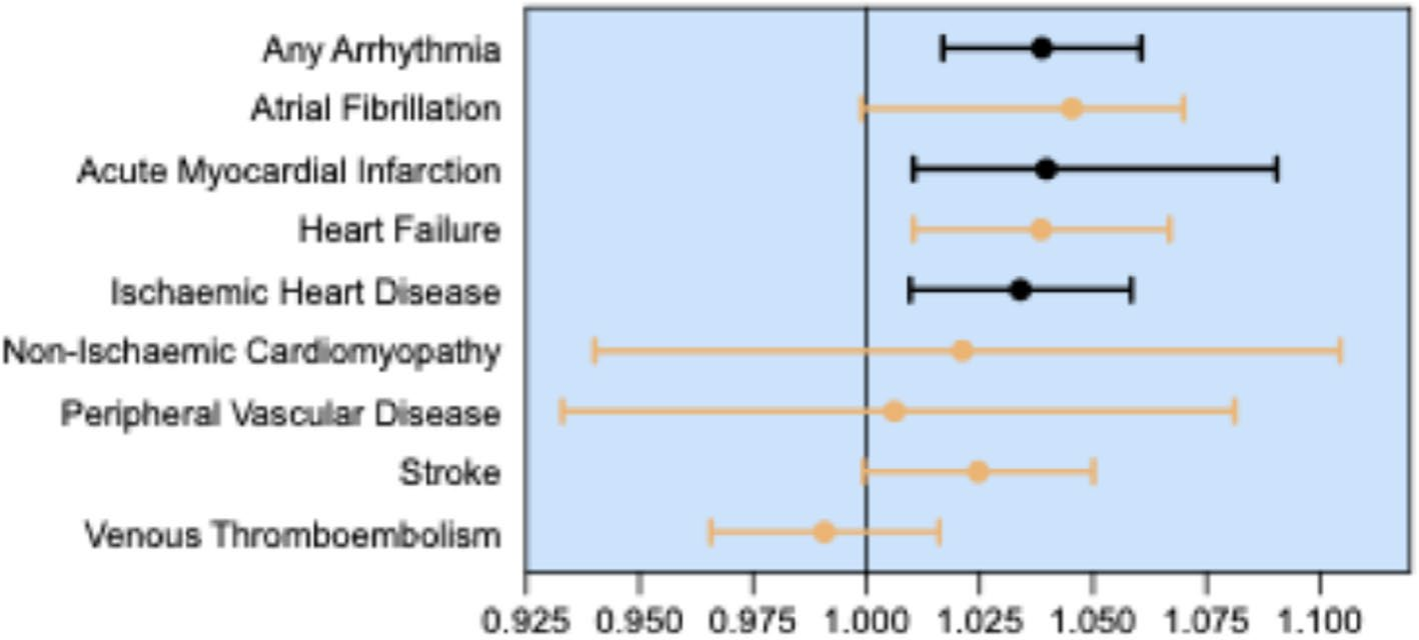
Associations between RA and CVD outcomes from MR analysis. Forest plot of Mendelian randomisation estimates of RA associations with CVD outcomes. The *x*-axis represents the odds ratio (OR) with the 95% confidence interval (CI). The *y*-axis lists the cardiovascular outcomes analysed: any arrhythmia, atrial fibrillation, acute myocardial infarction, heart failure, ischaemic heart disease, non-ischaemic cardiomyopathy, peripheral vascular disease, stroke and venous thromboembolism. The orange bars indicate statically nonsignificant associations

IVW analysis revealed a significant causal association between genetically predicted RA and AMI, IHD, any arrhythmia, AF, and HF. After correction for multiple tests and considering complementary analyses, three associations remained significant: AMI (OR = 1.07 [95% CI = 1.018, 1.090], corrected *p*-value = 0.009), any arrhythmia (OR = 1.0463 [95% CI = 1.016, 1.060], corrected *p*-value = 0.0007) and IHD (OR = 1.0456 [95% CI = 1.009, 1.058], corrected *p*-value = 0.036). The results of the MR-Egger weighted median and weighted mode analysis for these conditions were also consistent with those of the IVW method. MR-PRESSO global test (GT) suggested the existence of horizontal pleiotropy in the three associations (*p* < 0.001). After correcting for pleiotropy, these remained significant (distortion test *p* > 0.05). The intercept from the MR-Egger analysis did not indicate directional horizontal pleiotropy for AMI, IHD, or arrhythmia (*p* > 0.05). Comprehensive MR data regarding the association between RA and CVD are presented in Additional File 1: Tables 9 and 12 as well as in Fig. 3.

#### East Asian Mendelian randomisation analysis

A total of 43 independent genome-wide significant SNPs were used as genetic instruments for RA. Using IVW analysis, we observed nominal associations of genetically predicted RA with HF (*p* = 0.023), IHD (*p* = 0.027), and AMI (*p* = 0.113). These results were supported by complementary MR methods (e.g. MR Egger, weighted median and weighted mode), with MR-PRESSO identifying pleiotropy for IHD and AMI. However, after Bonferroni correction, no associations remained statistically significant (α < 0.00625). Full results are reported in Additional File 1: Tables 13 and 14.

### Relationship between RA and CMR measures

#### Association of RA with CMR metrics

Results for baseline CMR metrics stratified by RA status are presented in Additional File 1: Table 15. Participants with RA had slightly larger left and right atrial maximal volumes than controls. Similarly, the LVWT and global LVM were also marginally greater in those without RA. Left ventricular functional metrics were nearly identical between groups. In RA participants, RVEDV was slightly larger and RVEF was marginally lower than in controls. In fully adjusted models, no statistically significant associations were observed between RA and any of the CMR metrics considered (Additional File 1: Table 16).

#### Causal association between RA and CMR metrics

The IVW analysis of CMR metrics indicated causal associations between RA and seven CMR metrics (Additional File 1: Table 17). However, after correcting for multiple tests, only four metrics (RVESV, RVEDV, RVEF and LVM) remained significant. Complementary analyses using MR Egger and weighted median did not support the significance of these associations. MR-PRESSO identified horizontal pleiotropy in two metrics (RVESV and RVEDV); after correction, none were significant. The Egger intercept test for the four associations was not significant (*p* > 0.05), indicating no directional horizontal pleiotropy. Detailed results are presented in Additional File 1: Table 17 and Additional File 2: Fig. 1.

## Discussion

### Summary of findings

This study conducted a comprehensive investigation into the relationship between RA and cardiovascular health using a large-scale biomedical database integrating clinical health records, cardiovascular imaging, and genetic information. Our triangulated analytic approach—incorporating observational associations, genetic causal analyses, and CMR imaging—enabled a multidimensional assessment of cardiovascular risk in RA. We observed that RA is associated with a significantly higher prevalence and incidence of multiple CVDs, including less commonly reported outcomes such as pericardial disease and non-ischaemic cardiomyopathies. These findings broaden the spectrum of cardiovascular outcomes associated with RA and extend previous literature. Additionally, MR analyses suggest the causal relevance of relationships between RA and ischaemic CVDs and arrhythmias. We found no association between RA and CMR-derived measures, indicating that conventional CMR parameters may not capture subclinical myocardial involvement in unselected RA populations and help define the boundaries of CMR utility in this context.

These findings provide insight into mechanistic pathways underlying the RA-CVD relationship and support efforts towards improved long-term cardiovascular care and risk stratification strategies.

### Comparison with existing literature

Our observational analyses demonstrated a significantly elevated risk of incident AMI and IHD in RA patients over 14 years of follow-up, independent of traditional cardiovascular risk factors. These results align with prior studies, reporting increased coronary plaque burden, silent AMI, and sudden cardiac death in RA populations [43–45]. Notably, the risk of AMI was particularly pronounced in Rheumatoid Factor-positive patients and those with high disease activity (DAS28 ≥ 3.2) [44]. Our MR analyses provide novel causal evidence linking RA to IHD and AMI, confirming that RA directly increases the risk of IHD. These results are consistent with those reported by Wang et al. [16], further supporting the robustness of our findings. Our study provides a substantially larger sample size, with 22,350 RA cases and 74,823 controls. Thus, our results corroborate findings of smaller observational studies in a much larger population-based sample and present genetic evidence further supporting the causality of the relationship between RA and ischaemic CVDs. Our analysis additionally demonstrated a higher incidence of PVD.

While RA is integrated into existing CVD risk algorithms aimed at predicting the risk of IHD and stroke [45], strategies targeting non-standard CVD pathways are needed to reduce the substantial cardiovascular burden in this vulnerable population. Further studies are needed to uncover the distinct biologic pathways driving IHD in the context of RA, and potential targeted therapeutics that may be relevant to this population. In terms of assessment for pre-test clinical likelihood for obstructive atherosclerotic CAD, as recommended in current guidelines [46], individualised adjustments may be necessary for RA patients. However, specific recommendations, such as earlier use of non-invasive testing for CAD, are yet to be implemented and may be considered. Additionally, the cardiovascular effects of DMARDs should be considered when evaluating cardiovascular risk in RA patients. Systematic reviews suggest that biologic therapies, including TNF inhibitors, decrease CVD morbidity, despite modest increases in total cholesterol and triglyceride levels [47, 48]. IL-6 inhibitors may also offer cardiovascular protection through potent anti-inflammatory effects, although they have been reported to increase serum lipids [49]. Long-term corticosteroid use is associated with an adverse cardiometabolic profile, including increased blood pressure, insulin resistance, and dyslipidaemia, and may exacerbate CVD risk.

In our study, the risk of incident pericardial disease was almost 2.6-fold greater in participants with RA compared to controls. Autopsy studies have reported pericardial involvement in 30–50% of RA patients, highlighting its relatively high prevalence in this population. However, clinically apparent pericarditis is observed far less frequently, with an estimated prevalence of less than 10% [50]. Pericarditis, often associated with pericardial effusion, has been particularly noted in RA patients with severe, destructive, and nodular disease [50]. In some instances, these patients present with cardiac tamponade requiring urgent intervention [50].

This increased risk is likely driven by a dysregulated cytokine milieu, with pro-inflammatory mediators such as tumour necrosis factor-alpha (TNF-α), interleukin-1 (IL-1) and interleukin-6 (IL-6) promoting systemic inflammation and immune activation [51, 52]. Our study extends these observations and quantifies the risk in a large, unselected cohort. The long-term sequelae of the chronic untreated inflammatory pericardial disease comprise the development of constrictive physiology with a very poor associated prognosis and limited treatment options. These findings emphasise the need for proactive consideration of high-risk cardiovascular complications such as pericardial disease in patients with RA and a low threshold for investigation (e.g. echocardiography).

Arrhythmias represent another significant cardiovascular manifestation in RA, with our study revealing a substantially higher prevalence and incidence of AF and other arrhythmias among RA participants. Specifically, we observed a twofold increase in prevalent AF and a 24% higher risk of incident AF, even after adjusting for confounders. Importantly, our MR analyses provide new evidence indicating a likely causal relationship between RA and cardiac arrhythmias, reinforcing the hypothesis that RA directly contributes to arrhythmogenesis. Chronic systemic inflammation characteristic of RA, structural remodelling, and autonomic dysfunction in RA patients likely create a proarrhythmic environment [19, 53].

Other MR studies investigating the relationship between RA and arrhythmias are limited and inconsistent. For instance, Wang et al. [16] and Song et al. [20] found no causal link between RA and arrhythmia and AF. However, two recent studies [19, 54] demonstrated a causal link, using GWAS datasets derived from Asian ancestry populations. Our study, which utilised European ancestry RA GWAS, provides new robust evidence supporting this causal relationship and broadens its generalisability. These findings highlight the importance of focused rhythm assessments and tailored management strategies to address arrhythmia-related risks in this high-risk population to mitigate related complications, such as thromboembolic strokes. Indeed, the integration of opportunistic pulse checks, electrocardiograms (ECGs) and wearable cardiac monitoring devices is likely a worthwhile addition to the holistic assessment of RA patients.

To explore the generalisability of our findings across different ethnicities, we performed additional MR analysis using GWAS summary statistics from East Asian populations. While the direction of associations for outcomes such as HF, IHD, and AMI was broadly consistent with European findings, statistical significance was not reached after multiple testing correction. Several factors may contribute to these differences. First, analytical variation across studies—such as differences in variant inclusion criteria, rare variant burden testing versus common variant GWAS—can affect effect size estimation and comparability. Second, population-specific genetic architecture plays a major role: allele frequencies, effect sizes, and linkage disequilibrium patterns differ between ancestries, directly influencing the validity and strength of genetic instruments used in MR. For example, founder effects in Finnish populations have led to enrichment of rare deleterious variants [30], while variant frequencies in East Asian populations frequently diverge from those observed in European cohorts [27]. These differences can complicate the identification and interpretation of causal variants, as also demonstrated by Kanai et al. in their multi-ethnic fine-mapping study [55]. In addition, differences may be due to limitations caused by smaller sample sizes and lower disease prevalence in the East Asian datasets.

In fully adjusted model, patients with RA had 53% greater odds of hypertension. While the risk of these conditions is likely amplified by shared risk factors (obesity, smoking, sedentary lifestyle), our results indicate a role for RA-specific contributors such as chronic inflammation, immune system dysfunction and reduced physical activity due to pain and disability [56–59]. The widespread use of glucocorticoids and nonsteroidal anti-inflammatory drugs (NSAIDs) exacerbates this risk, with glucocorticoids exerting both hypertensive and diabetogenic effects [60]. These findings emphasise the importance of proactive screening and control of cardiometabolic risk conditions in RA patients. The current ESC hypertension guidelines identify inflammatory conditions like RA as non-traditional cardiovascular risk modifiers. They recommend up-classifying individuals with RA, especially those on systemic therapies or with significant disease burden, to higher risk categories [61]. Incorporating blood pressure and glucose monitoring into RA care pathways, along with patient education on lifestyle factors, could significantly reduce long-term cardiovascular complications. Additionally, encouraging home blood pressure monitoring could be an effective strategy to improve adherence and enable earlier intervention.

Lastly, our study investigated the relationship between RA and CMR metrics using observational and MR analysis with large-scale GWAS data. Key findings include (1) RA was associated with marginally higher left and right atrial volumes, greater LVM, and slightly poorer RV function compared to controls; however, these relationships were not statistically significant in fully adjusted models; (2) MR analysis found no causal link between genetically predicted RA and CMR metrics considered. While advanced CMR techniques such as native T1 mapping, extracellular volume fraction (ECV) and T2 mapping have shown subclinical myocardial changes in RA patients [11–13], these methods are resource-intensive. Our findings do not support a role for the routine use of CMR in unselected cohorts of patients with RA and suggest that standard structural and functional CMR parameters may not detect early myocardial changes in asymptomatic or unselected RA populations. CMR should be reserved for patients with suspected cardiac disease based on clinical consultation with a cardiologist [62].

### Limitations

Potential confounding factors, such as unmeasured lifestyle influences, may have independently impacted cardiovascular outcomes in the observational analyses. Reliance on hospital records for outcome ascertainment may introduce selection bias by overrepresenting severe CVD cases and underestimating subclinical conditions. A more granular characterisation of RA severity was impossible using our dataset and may provide further insight into high-risk subgroups. Although we attempted to explore variation by RA treatment type, the number of participants receiving biologic therapy was small, resulting in insufficient power for stratified analyses. The MR analyses, based on participants of European ancestry, may limit generalisability to other ethnic groups. Although MR strengthens causal inference by reducing confounding and reverse causation, its validity depends on key assumptions—such as the absence of pleiotropy and correct specification of the genetic instruments—that may not always be fully verifiable. The use of summary-level GWAS data may also limit the detection of effects driven by rare variants or interactions between genetic loci. While standard CMR metrics showed no significant associations with RA, other sequences, for example, using contrast-enhanced imaging, were not available as part of the UK Biobank cohort and may warrant examination in future studies. Finally, the MR analysis in East Asian populations was limited by reduced sample sizes, lower disease prevalence, and differences in genetic architecture, which may have weakened instrument strength and statistical power. These limitations underscore the need for larger, ancestry-specific GWAS to support trans-ethnic causal inference. Our results should be interpreted in the context of these limitations. Although MR strengthens causal inference, it does not establish causality with certainty, and residual bias cannot be excluded. Findings may not be generalisable to non-European ancestry groups, and causal inference remains probabilistic rather than definitive.

### Recommendations for clinical practice

Our study underscores several practical clinical implications for reducing cardiovascular risk in patients with RA. A key recommendation is the need for a multidisciplinary cardio-rheumatology approach, which involves close collaboration between rheumatologists and cardiologists. This integrated model of care can facilitate early detection of cardiovascular complications, improve management strategies, and ultimately optimise outcomes for RA patients. Furthermore, it is critical to routinely evaluate the cardiovascular risk in RA patients, as their risk profile differs from the general population. Proactive screening of cardiometabolic conditions is also essential, given their increased prevalence in RA. Overall, our study highlights the importance of a proactive, comprehensive, and individualised approach to cardiovascular risk management in RA patients.

## Conclusions

This study provides robust evidence of the increased cardiovascular burden in RA patients, demonstrating higher risks of IHD, stroke, pericardial disease and AF. Through MR analysis, we established likely causal relationships between RA and key cardiovascular outcomes, further supporting the direct contribution of RA to cardiovascular disease. These results highlight the importance of routine screening, early intervention, and a multidisciplinary approach to care in order to reduce the elevated cardiovascular risk in this vulnerable population.

## Supplementary Information

Additional file 1: Table 1: Ascertainment of rheumatoid arthritis using UK Biobank fields and ICD codes. Table 2: Record of specific medications for rheumatoid arthritis. Table 3: Ascertainment of major metabolic morbidities. Table 4: Cardiovascular magnetic resonance imaging metrics used in the study, their clinical definition and interpretation. Table 5: Summary of GWAS for cardiovascular conditions Table 6: Summary of GWAS for CMR metrics. Table 7: Characteristics of genetic variants as instrumental variables (IVs) Table 8: Summary of GWAS for cardiovascular conditions in East Asian populations. Table 9: Power calculation for Mendelian Randomisation analysis of RA and CVD. Table 10: Non-MICE model for the association of rheumatoid arthritis with prevalent cardiovascular diseases and vascular risk factors. Table 11: Non-MICE model for the association of rheumatoid arthritis with incident cardiovascular diseases. Table 12: MR estimates of rheumatoid arthritis associated with the risk of cardiovascular diseases. Table 13: Characteristics of genetic variants used as instrumental variables (IVs) in the East Asian Mendelian Randomisation analysis. Table 14: MR estimates of rheumatoid arthritis associated with the risk of cardiovascular diseases in an East Asian population. Table 15: Baseline CMR comparison between participants without rheumatoid arthritis (No RA) and with rheumatoid arthritis RA. Table 16: Association of rheumatoid arthritis with CMR metrics. Table 17: MR estimates of rheumatoid arthritis and CMR metrics

Additional file 2. Figure 1: Associations between RA and CMR metrics

## Data Availability

This project was conducted under UK Biobank Access Application 3593. All researchers must follow the same application process and meet the approval criteria outlined by UK Biobank. For details on the data access procedure, visit the UK Biobank website [63]. [https://www.ukbiobank.ac.uk/register-apply](https://www.ukbiobank.ac.uk/register-apply). Summary statistics for cardiovascular outcomes were obtained from the FinnGen study(FinnGen Data Freeze R10, 2023), accessible at [https://www.finngen.fi/en](https://www.finngen.fi/en?utm_source=chatgpt.com) [64]. Summary statistics for East Asian outcomes were obtained from the BioBank Japan Project via PheWeb Japan (NBDC/JST), available at [https://pheweb.jp](https://pheweb.jp) [65].

## Abbreviations

RA: Rheumatoid arthritis
CVD: Cardiovascular disease
MR: Mendelian randomisation
GWAS: Genome-wide association study
IVW: Inverse variance-weighted
CMR: Cardiovascular magnetic resonance
IHD: Ischaemic heart disease
AMI: Acute myocardial infarction
AF: Atrial fibrillation
HF: Heart failure
NICM: Non-ischaemic cardiomyopathy
PVD: Peripheral vascular disease
VTE: Venous thromboembolism
