# Supplementary material for: Rheumatoid arthritis and cardiovascular disease associations in the UK Biobank
Source: BMC Med. 2025 Nov 3;23:605. doi: 10.1186/s12916-025-04431-1 (PMC12581239; doi:10.1186/s12916-025-04431-1)
Supplement: Supplementary file 1 — Additional file 1: Table 1: Ascertainment of rheumatoid arthritis using UK Biobank fields and ICD codes. Table 2: Record of specific medications for rheumatoid arthritis. Table 3: Ascertainment of major metabolic morbidities. Table 4: Cardiovascular magnetic resonance imaging metrics used in the study, their clinical definition and interpretation. Table 5: Summary of GWAS for cardiovascular conditions Table 6: Summary of GWAS for CMR metrics. Table 7: Characteristics of genetic variants as instrumental variables (IVs) Table 8: Summary of GWAS for cardiovascular conditions in East Asian populations. Table 9: Power calculation for Mendelian Randomisation analysis of RA and CVD. Table 10: Non-MICE model for the association of rheumatoid arthritis with prevalent cardiovascular diseases and vascular risk factors. Table 11: Non-MICE model for the association of rheumatoid arthritis with incident cardiovascular diseases. Table 12: MR estimates of rheumatoid arthritis associated with the risk of cardiovascular diseases. Table 13: Characteristics of genetic variants used as instrumental variables (IVs) in the East Asian Mendelian Randomisation analysis. Table 14: MR estimates of rheumatoid arthritis associated with the risk of cardiovascular diseases in an East Asian population. Table 15: Baseline CMR comparison between participants without rheumatoid arthritis (No RA) and with rheumatoid arthritis RA. Table 16: Association of rheumatoid arthritis with CMR metrics. Table 17: MR estimates of rheumatoid arthritis and CMR metrics [file 12916_2025_4431_MOESM1_ESM.docx]

Additional file 1

Table of content

[Table 1: Ascertainment of rheumatoid arthritis using UK Biobank fields and ICD codes 2](#_Toc209975373)

[Table 2: Record of specific medications for rheumatoid arthritis 5](#_Toc209975374)

[Table 3: Ascertainment of major metabolic morbidities 6](#_Toc209975375)

[Table 4: Cardiovascular magnetic resonance imaging metrics used in the study, their clinical definition and interpretation 13](#_Toc209975376)

[Table 5: Summary of GWAS for cardiovascular conditions 14](#_Toc209975377)

[Table 6: Summary of GWAS for CMR metrics 15](#_Toc209975378)

[Table 7: Characteristics of genetic variants as instrumental variables (IVs) 16](#_Toc209975379)

[Table 8: Summary of GWAS for cardiovascular conditions in East Asian populations 18](#_Toc209975380)

[Table 9: Power calculation for Mendelian Randomisation analysis of RA and CVD 19](#_Toc209975381)

[Table 10: Non-MICE Model for the Association of Rheumatoid Arthritis with Prevalent Cardiovascular Diseases and Vascular Risk Factors 20](#_Toc209975382)

[Table 11: Non-MICE Model for the Association of Rheumatoid Arthritis with Incident Cardiovascular Diseases 21](#_Toc209975383)

[Table 12: MR estimates of rheumatoid arthritis associated with the risk of cardiovascular diseases 22](#_Toc209975384)

[Table 13: Characteristics of genetic variants used as instrumental variables (IVs) in the East Asian Mendelian Randomisation analysis 23](#_Toc209975385)

[Table 14: MR estimates of rheumatoid arthritis associated with the risk of cardiovascular diseases in an East Asian population 25](#_Toc209975386)

[Table 15: Baseline Cardiac Magnetic Resonance Metrics Comparison Between Participants Without Rheumatoid Arthritis (No RA) and With Rheumatoid Arthritis (RA) 26](#_Toc209975387)

[Table 16: Association of Rheumatoid Arthritis with Cardiac Magnetic Resonance Metrics 27](#_Toc209975388)

[Table 17: MR estimates of rheumatoid arthritis and CMR metrics 28](#_Toc209975389)

# Table 1: Ascertainment of rheumatoid arthritis using UK Biobank fields and ICD codes

| **Diagnostic label** | **UKB Field/ ICD code** | **Code/label** | **Notes** |
| --- | --- | --- | --- |
| Rheumatoid arthritis | 20002 | "rheumatoid arthritis” |  |
|  | 120001 | If “Yes” take as Positive for rheumatoid arthritis status | Answer to question “Ever had rheumatoid arthritis affecting one or more joints?” |
|  | ICD10 | M05.00 Felty's syndrome (Multiple sites)  M05.09 Felty's syndrome (Site unspecified)  M05.10 Rheumatoid lung disease (Multiple sites)  M05.17 Rheumatoid lung disease (Ankle and foot)  M05.18 Rheumatoid lung disease (Other)  M05.19 Rheumatoid lung disease (Site unspecified)  M05.20 Rheumatoid vasculitis (Multiple sites)  M05.24 Rheumatoid vasculitis (Hand)  M05.26 Rheumatoid vasculitis (Lower leg)  M05.28 Rheumatoid vasculitis (Other)  M05.29 Rheumatoid vasculitis (Site unspecified)  M05.30 Rheumatoid arthritis with involvement of other organs and systems  M05.38 Rheumatoid arthritis with involvement of other organs and systems (Other)  M05.39 Rheumatoid arthritis with involvement of other organs and systems (Site unspecified)  M05.80 Other seropositive rheumatoid arthritis (Multiple sites)  M05.82 Other seropositive rheumatoid arthritis (Upper arm)  M05.83 Other seropositive rheumatoid arthritis (Forearm)  M05.84 Other seropositive rheumatoid arthritis (Hand)  M05.86 Other seropositive rheumatoid arthritis (Lower leg)  M05.87 Other seropositive rheumatoid arthritis (Ankle and foot)  M05.88 Other seropositive rheumatoid arthritis (Other)  M05.89 Other seropositive rheumatoid arthritis (Site unspecified)  M05.90 Seropositive rheumatoid arthritis, unspecified (Multiple sites)  M05.91 Seropositive rheumatoid arthritis, unspecified (Shoulder region)  M05.92 Seropositive rheumatoid arthritis, unspecified (Upper arm)  M05.93 Seropositive rheumatoid arthritis, unspecified (Forearm)  M05.94 Seropositive rheumatoid arthritis, unspecified (Hand)  M05.95 Seropositive rheumatoid arthritis, unspecified (Pelvic region and thigh)  M05.96 Seropositive rheumatoid arthritis, unspecified (Lower leg)  M05.97 Seropositive rheumatoid arthritis, unspecified (Ankle and foot)  M05.98 Seropositive rheumatoid arthritis, unspecified (Other)  M05.99 Seropositive rheumatoid arthritis, unspecified (Site unspecified)  M06.00 Seronegative rheumatoid arthritis (Multiple sites)  M06.01 Seronegative rheumatoid arthritis (Shoulder region)  M06.02 Seronegative rheumatoid arthritis (Upper arm)  M06.03 Seronegative rheumatoid arthritis (Forearm)  M06.04 Seronegative rheumatoid arthritis (Hand)  M06.05 Seronegative rheumatoid arthritis (Pelvic region and thigh)  M06.06 Seronegative rheumatoid arthritis (Lower leg)  M06.07 Seronegative rheumatoid arthritis (Ankle and foot)  M06.08 Seronegative rheumatoid arthritis (Other)  M06.09 Seronegative rheumatoid arthritis (Site unspecified)  M06.21 Rheumatoid bursitis (Shoulder region)  M06.22 Rheumatoid bursitis (Upper arm)  M06.25 Rheumatoid bursitis (Pelvic region and thigh)  M06.26 Rheumatoid bursitis (Lower leg)  M06.27 Rheumatoid bursitis (Ankle and foot)  M06.30 Rheumatoid nodule (Multiple sites)  M06.32 Rheumatoid nodule (Upper arm)  M06.33 Rheumatoid nodule (Forearm)  M06.34 Rheumatoid nodule (Hand)  M06.36 Rheumatoid nodule (Lower leg)  M06.37 Rheumatoid nodule (Ankle and foot)  M06.38 Rheumatoid nodule (Other)  M06.39 Rheumatoid nodule (Site unspecified)  M06.80 Other specified rheumatoid arthritis (Multiple sites)  M06.81 Other specified rheumatoid arthritis (Shoulder region)  M06.82 Other specified rheumatoid arthritis (Upper arm)  M06.84 Other specified rheumatoid arthritis (Hand)  M06.85 Other specified rheumatoid arthritis (Pelvic region and thigh)  M06.86 Other specified rheumatoid arthritis (Lower leg)  M06.87 Other specified rheumatoid arthritis (Ankle and foot)  M06.88 Other specified rheumatoid arthritis (Other)  M06.89 Other specified rheumatoid arthritis (Site unspecified)  M06.90 Rheumatoid arthritis, unspecified (Multiple sites)  M06.91 Rheumatoid arthritis, unspecified (Shoulder region)  M06.92 Rheumatoid arthritis, unspecified (Upper arm)  M06.93 Rheumatoid arthritis, unspecified (Forearm)  M06.94 Rheumatoid arthritis, unspecified (Hand)  M06.95 Rheumatoid arthritis, unspecified (Pelvic region and thigh)  M06.96 Rheumatoid arthritis, unspecified (Lower leg)  M06.97 Rheumatoid arthritis, unspecified (Ankle and foot)  M06.98 Rheumatoid arthritis, unspecified (Other)  M06.99 Rheumatoid arthritis, unspecified (Site unspecified) |  |
|  | 131848 | If any valid date entered, take as Positive for rheumatoid arthritis status and as date of first occurrence | Date M05 first reported (seropositive rheumatoid arthritis) |
|  | 131850 | If any valid date entered, take as Positive for rheumatoid arthritis status and as date of first occurrence | Date M06 first reported (other rheumatoid arthritis) |
|  | ICD9 | 71400 Rheumatoid arthritis (multiple sites)  71401 Rheumatoid arthritis (shoulder region)  71403 Rheumatoid arthritis (forearm)  71404 Rheumatoid arthritis (hand)  71405 Rheumatoid arthritis (pelvic region and thigh)  71406 Rheumatoid arthritis (lower leg)  71409 Rheumatoid arthritis (site unspecified)  71416 Felty's syndrome (lower leg) |  |

**Footnote Table 1:** RA status at baseline recruitment was determined using self-reported diagnoses, medication records, and linked hospital data. Supplementary Table 1 details the UK Biobank field IDs and ICD-9/ICD-10 codes used to define rheumatoid arthritis cases. Participants were classified as RA-positive if they had a relevant diagnosis code or self-reported RA, and only if this was supported by the use of RA-specific medications.

# Table 2: Record of specific medications for rheumatoid arthritis

| Systemic steroids | Corticosteroid, Methylprednisolone, Medrone, Prednisone, Deltacortril, Deltastab, Precortisyl, Prednesola |
| --- | --- |
| Gold Salt | Auranofin, Ridaura |
| Conventional DMARD | Azathioprine, Imuran, Hydroxychloroquine, Plaquenil, Myocrisin, Sodium Aurothiomalate, Sulfasalazine, Sulphasalazine, Sulazine |
| Biologic DMARD | Adalimumab, Humira, Anakinra, Kineret |

**Footnote Table 2:** List of rheumatoid arthritis-specific medications considered for case classification. Medications were grouped as systemic steroids, gold salts, conventional disease-modifying antirheumatic drugs (DMARDs), and biologic DMARDs. Presence of any listed medication was used to support RA diagnosis classification. DMARD - Disease-Modifying Antirheumatic Drug

# Table 3: Ascertainment of major metabolic morbidities

| **Diagnostic label** | **UKB Field/ICD code** | **Code/label** | **Notes** |
| --- | --- | --- | --- |
| Diabetes | 20002 | “type 1 diabetes” |  |
|  |  | “type 2 diabetes” |  |
|  | 6177 | "medication for diabetes” | Insulin |
|  | 6153 | “medication for diabetes” | Insulin |
|  | 6148 | “diabetes related eye disease” | Answer to question “Has a doctor told you that you have any of the following problems with your eyes?” |
|  | 2443 | If “Yes” take as Positive for diabetes status | Answer to question "Has a doctor ever told you that you have diabetes?" |
|  | 120007 | If “Yes” take as Positive for diabetes status | Answer to question “Ever had diabetes (Type I or Type II)?” |
|  | 2976 | If any valid age entered, take as Positive for diabetes status and take the age as date of first occurrence | Answer to question ""What was your age when the diabetes was first diagnosed?" |
|  | 5901 | If any valid age entered, take as Positive for diabetes status and take the age as date of first occurrence | Answer to question ""What was your age when the diabetes related eye disease was first diagnosed?" |
|  | 30750 | If HbA1c >48 mmol/mol take as Positive for diabetes status |  |
|  | 30740 | If serum glucose >11.1 mmol/L take as Positive for diabetes status |  |
|  | ICD10 (primary or secondary diagnosis), HES or death register | E10 Insulin-dependent diabetes mellitus- |  |
|  |  | E10.0 With coma |  |
|  |  | E10.1 With ketoacidosis |  |
|  |  | E10.2 With renal complications |  |
|  |  | E10.3 With ophthalmic complications |  |
|  |  | E10.4 With neurological complications |  |
|  |  | E10.5 With peripheral circulatory complications |  |
|  |  | E10.6 With other specified complications |  |
|  |  | E10.7 With multiple complications |  |
|  |  | E10.8 With unspecified complications |  |
|  |  | E10.9 Without complications |  |
|  |  | E11 Non-insulin-dependent diabetes mellitus- |  |
|  |  | E11.0 With coma |  |
|  |  | E11.1 With ketoacidosis |  |
|  |  | E11.2 With renal complications |  |
|  |  | E11.3 With ophthalmic complications |  |
|  |  | E11.4 With neurological complications |  |
|  |  | E11.5 With peripheral circulatory complications |  |
|  |  | E11.6 With other specified complications |  |
|  |  | E11.7 With multiple complications |  |
|  |  | E11.8 With unspecified complications |  |
|  |  | E11.9 Without complications |  |
|  |  | E12 Malnutrition-related diabetes mellitus- |  |
|  |  | E12.1 With ketoacidosis |  |
|  |  | E12.3 With ophthalmic complications |  |
|  |  | E12.5 With peripheral circulatory complications |  |
|  |  | E12.8 With unspecified complications |  |
|  |  | E12.9 Without complications |  |
|  |  | E13 Other specified diabetes mellitus- |  |
|  |  | E13.0 With coma |  |
|  |  | E13.1 With ketoacidosis |  |
|  |  | E13.2 With renal complications |  |
|  |  | E13.3 With ophthalmic complications |  |
|  |  | E13.4 With neurological complications |  |
|  |  | E13.5 With peripheral circulatory complications |  |
|  |  | E13.6 With other specified complications |  |
|  |  | E13.7 With multiple complications |  |
|  |  | E13.8 With unspecified complications |  |
|  |  | E13.9 Without complications |  |
|  |  | E14 Unspecified diabetes mellitus- |  |
|  |  | E14.0 With coma |  |
|  |  | E14.1 With ketoacidosis |  |
|  |  | E14.2 With renal complications |  |
|  |  | E14.3 With ophthalmic complications |  |
|  |  | E14.4 With neurological complications |  |
|  |  | E14.5 With peripheral circulatory complications |  |
|  |  | E14.6 With other specified complications |  |
|  |  | E14.7 With multiple complications |  |
|  |  | E14.8 With unspecified complications |  |
|  |  | E14.9 Without complications |  |
|  |  | O24.0 Pre-existing diabetes mellitus, insulin-dependent |  |
|  |  | O24.1 Pre-existing diabetes mellitus, noninsulin-dependent |  |
|  |  | O24.3 Pre-existing diabetes mellitus, unspecified |  |
|  | 130706 | If any valid date entered, take as Positive for diabetes status and as date of first occurrence | Date E10 first reported (insulin-dependent diabetes mellitus) |
|  | 130708 | If any valid date entered, take as Positive for diabetes status and as date of first occurrence | Date E11 first reported (non-insulin-dependent diabetes mellitus) |
|  | 130710 | If any valid date entered, take as Positive for diabetes status and as date of first occurrence | Date E12 first reported (malnutrition-related diabetes mellitus) |
|  | 130712 | If any valid date entered, take as Positive for diabetes status and as date of first occurrence | Date E13 first reported (other specified diabetes mellitus) |
|  | 130714 | If any valid date entered, take as Positive for diabetes status and as date of first occurrence | Date E14 first reported (unspecified diabetes mellitus) |
|  | ICD9 | 2500 Diabetes mellitus without mention of complication- |  |
|  |  | 25000 Diabetes mellitus without mention of complication (adult-onset type) |  |
|  |  | 25001 Diabetes mellitus without mention of complication (juvenile type) |  |
|  |  | 25009 Diabetes mellitus without mention of compl. (adult/juvenile unspec.) |  |
|  |  | 2501 Diabetes with ketoacidosis- |  |
|  |  | 25010 Diabetes with ketoacidosis (adult-onset type) |  |
|  |  | 25011 Diabetes with ketoacidosis (juvenile type) |  |
|  |  | 25019 Diabetes with ketoacidosis (adult/juvenile unspec.) |  |
|  |  | 2502 Diabetes with coma- |  |
|  |  | 25029 Diabetes with coma (unspecified whether adult-onset or juvenile type) |  |
|  |  | 2503 Diabetes with renal manifestations |  |
|  |  | 2504 Diabetes with ophthalmic manifestations |  |
|  |  | 2505 Diabetes with neurological manifestations |  |
|  |  | 2509 Diabetes with unspecified complications- |  |
|  |  | 25099 Diabetes with unspecified complications (unspecified onset) |  |
| Hypercholesterolaemia | 20002 | "high cholesterol” |  |
|  | 6177 | “medication for cholesterol” | Cholesterol lowering medication |
|  | 6153 | “medication for cholesterol” | Cholesterol lowering medication |
|  | ICD10 (primary or secondary diagnosis), HES or death register | E78.0 Pure hypercholesterolaemia |  |
|  |  | E78.1 Pure hyperglyceridaemia |  |
|  |  | E78.2 Mixed hyperlipidaemia |  |
|  |  | E78.3 Hyperchylomicronaemia |  |
|  |  | E78.4 Other hyperlipidaemia |  |
|  |  | E78.5 Hyperlipidaemia, unspecified |  |
|  | 130814 | If any valid date entered for E78.0, E78.1, E78.2, E78.3, E78.4 or E78.5, take as Positive for high cholesterol status and as date of first occurrence | Date E78.0, E78.1, E78.2, E78.3, E78.4 or E78.5 first reported (pure hypercholesterolaemia, pure hyperglyceridaemia, mixed hyperlipidaemia, hyperchylomicronaemia, other hyperlipidaemia or |
|  |  |  | hyperlipidaemia, unspecified) |
|  | ICD9 | 27200 Familial hypercholesterolaemia |  |
|  |  | 27209 Pure hypercholesterolaemia (other) |  |
|  |  | 2721 Pure hyperglyceridaemia |  |
|  |  | 2722 Mixed hyperlipidaemia |  |
|  |  | 27249 Hyperlipidaemia not otherwise specified |  |
| Hypertension | 20002 | "essential hypertension” |  |
|  | 6177 | “medication for blood pressure” | Blood pressure medication |
|  | 6153 | “medication for blood pressure” | Blood pressure medication |
|  | 6150 | “high blood pressure” | Answer to question “Has a doctor ever told you that you have had high blood pressure?” |
|  | 2966 | If any valid age entered, take as Positive for hypertension status and take the age as date of first occurrence | Answer to question “What was your age when the high blood pressure was first diagnosed?” |
|  | ICD10 (primary or secondary diagnosis), HES or death register | I10 Essential (primary) hypertension |  |
|  |  | I11.0 Hypertensive heart disease with (congestive) heart failure |  |
|  |  | I11.9 Hypertensive heart disease without (congestive) heart failure |  |
|  |  | I12.0 Hypertensive renal disease with renal failure |  |
|  |  | I12.9 Hypertensive renal disease without renal failure |  |
|  |  | I13.0 Hypertensive heart and renal disease with (congestive) heart failure |  |
|  |  | I13.1 Hypertensive heart and renal disease with renal failure |  |
|  |  | I13.2 Hypertensive heart and renal disease with both (congestive) heart failure and renal failure |  |
|  |  | I13.9 Hypertensive heart and renal disease, unspecified |  |
|  |  | I15.0 Renovascular hypertension |  |
|  |  | I15.1 Hypertension secondary to other renal disorders |  |
|  |  | I15.2 Hypertension secondary to endocrine disorders |  |
|  |  | I15.8 Other secondary hypertension |  |
|  |  | I15.9 Secondary hypertension, unspecified |  |
|  |  | O10.0 Pre-existing essential hypertension complicating pregnancy, childbirth and the puerperium |  |
|  |  | O10.1 Pre-existing hypertensive heart disease complicating pregnancy, childbirth and the puerperium |  |
|  |  | O10.2 Pre-existing hypertensive renal disease complicating pregnancy, childbirth and the puerperium |  |
|  |  | O10.9 Unspecified pre-existing hypertension complicating pregnancy, childbirth and the puerperium |  |
|  |  | O11 Pre-existing hypertensive disorder with superimposed proteinuria |  |
|  | 131286 | If any valid date entered, take as Positive for hypertension status and as date of first occurrence | Date I10 first reported (essential (primary) hypertension) |
|  | 131288 | If any valid date entered, take as Positive for hypertension status and as date of first occurrence | Date I11 first reported (hypertensive heart disease) |
|  | 131290 | If any valid date entered, take as Positive for hypertension status and as date of first occurrence | Date I12 first reported (hypertensive renal disease) |
|  | 131292 | If any valid date entered, take as Positive for hypertension status and as date of first occurrence | Date I13 first reported (hypertensive heart and renal disease) |
|  | 131294 | If any valid date entered, take as Positive for hypertension status and as date of first occurrence | Date I15 first reported (secondary hypertension) |
|  | 132180 | If any valid date entered, take as Positive for hypertension status and as date of first occurrence | Date O10 first reported (pre-existing hypertension complicating pregnancy, childbirth and the puerperium) |
|  | 132182 | If any valid date entered, take as Positive for hypertension status and as date of first occurrence | Date O11 first reported (pre-existing hypertensive disorder with superimposed proteinuria) |
|  | ICD9 | 4010 Essential hypertension, specified as malignant |  |
|  |  | 4011 Essential hypertension, specified as benign |  |
|  |  | 4019 Essential hypertension, not specified as malignant or benign |  |
|  |  | 4039 Hypertensive renal disease, not specified as malignant or benign |  |
|  |  | 6427 Pre-eclampsia or eclampsia superimposed on pre-existing hypertension |  |

**Footnote Table 3:** Definition of diagnostic labels was based on self-reported data, medication records, clinical measurements, and linked hospital episode statistics using ICD-9 and ICD-10 codes. A condition was considered present if any of the listed criteria were met, including diagnosis codes, relevant medication usage, biomarker thresholds, or affirmative questionnaire responses. For selected variables (e.g., date of first occurrence), age or calendar date entries were used if available. ICD-10 and ICD-9 codes were obtained from UK Biobank hospital and death records.

# Table 4: Cardiovascular magnetic resonance imaging metrics used in the study, their clinical definition and interpretation

| **CMR metrics** | **Measurement Overview** | **Calculation** |
| --- | --- | --- |
| LV WT Global (mm) | LV myocardial wall thickness (mm), measured on short axis stack. | Reported as global mean thickness and per AHA segments |
| LVM (g) | Estimated total mass of the LV myocardium from short axis stack images. | Myocardial volume * density (1.05 g/mL) |
| LVEDV (mL) | A measure of LV size. | LV end-diastolic volume from short axis stack |
| LVM : LVEDV (g/mL) | The ratio of LV mass to LV end-diastolic volume. This gives a measure of the pattern of LV hypertrophy. | The ratio of LVM/LVEDV |
| LVSV (mL) | The volume ejected by LV in one cardiac cycle, a measure of LV function. | LVEDV-LVESV |
| LVEF (%) | The volume ejected by the LV in one cardiac cycle as percentage of total volume in the LV, a widely used measure of LV function. | (LVEDV-LVESV)/LVEDV x100 |
| LVGFI (%)* | A measure of LV function which takes into account cardiac structure. | LVGFI (%) as LV stroke volume/LV global volume x 100, where LV global volume was calculated as the sum of the LV mean cavity volume [(LV end-diastolic volume + LV end- systolic volume)/2] and myocardium volume (LV mass/density). Density = 1.05 g/mL |
| LV GLS (%) | A measure of LV function, estimating longitudinal deformation or shortening of the LV | Peak longitudinal strain for each segment on the long-axis view and global peak longitudinal strain. |
| RVEDV (mL) | A measure of RV size | End-diastolic volume from short axis stack |
| RVSV (mL) | A measure of RV function | (RVEDV - RVESV) |
| RVEF (%) | A measure of RV function | (RVEDV - RVESV) / RVEDV x 100 |
| AoD (10^-3^ mmHg^-1^) | A measure of local aortic compliance | (DAo Max Area - DAo Min Area) / (Dao Min Area * (SBP - DBP)) |

**Footnote Table 4:** **Cardiovascular magnetic resonance metrics** included in this study encompassed structural and functional parameters of the left and right ventricles, as well as aortic distensibility. AoD – aortic distensibility at the proximal descending aorta; CMR – cardiovascular magnetic resonance; GLS – global longitudinal strain; LV – left ventricle; LVEDV – left ventricular end-diastolic volume; LVEF – left ventricular ejection fraction; LVGFI – left ventricular global function index; LV GLS – left ventricular global longitudinal strain; LVM – left ventricular mass; LVSV – left ventricular stroke volume; LV WT – left ventricular wall thickness; RV – right ventricle; RVEDV – right ventricular end-diastolic volume; RVEF – right ventricular ejection fraction; RVSV – right ventricular stroke volume.

.

# Table 5: Summary of GWAS for cardiovascular conditions

| **Outcome** | **Reference (paper)** | **Cohort** | **Cases** | **Control** | **Sample size** | **GWAS summary statistics link** |
| --- | --- | --- | --- | --- | --- | --- |
| Ischaemic heart  disease | Kurki MI et al. ^1^ | FinnGen | 69,008 | 343,173 | 412,181 | <https://r10.finngen.fi/pheno/I9_IHD> |
| Acute myocardial  infarction |  |  | 26,060 | 343,079 | 369,139 | <https://r10.finngen.fi/pheno/I9_MI_STRICT> |
| Heart failure |  |  | 29,672 | 382,509 | 412,181 | <https://r10.finngen.fi/pheno/I9_HEARTFAIL> |
| Atrial fibrillation |  |  | 50,743 | 210,652 | 261,395 | <https://r10.finngen.fi/pheno/I9_AF> |
| Any arrhythmia |  |  | 74,000 | 239,778 | 313,778 | <https://r10.finngen.fi/pheno/CARDIAC_ARRHYTM> |
| Non-ischaemic  cardiomyopathy |  |  | 1,754 | 340,815 | 342,569 | <https://r10.finngen.fi/pheno/I9_NONISCHCARDMYOP_STRICT> |
| Stroke |  |  | 43,132 | 297,867 | 340,999 | <https://r10.finngen.fi/pheno/C_STROKE> |
| Peripheral vascular  disease |  |  | 2,489 | 381,977 | 384,466 | <https://r10.finngen.fi/pheno/I9_OTHPER> |
| Venous  thromboembolism |  |  | 21,021 | 391,160 | 412,181 | <https://r10.finngen.fi/pheno/I9_VTE> |

**Footnote Table 5:** The table summarises genome-wide association studies (GWAS) used to define cardiovascular outcomes in the Mendelian randomisation analysis. For each condition, the table lists the cohort, number of cases and controls, total sample size, and direct link to the publicly available summary statistics. All GWAS were conducted within the FinnGen study (R10 release). GWAS - genome wide association studies.

^1^ Kurki MI, Karjalainen J, Palta P, Sipilä TP, Kristiansson K, Donner K, et al. FinnGen: Unique genetic insights from combining isolated population and national health register data. medRxiv. 2022;:2022.03.03.22271360.

# Table 6: Summary of GWAS for CMR metrics

| **CMR metrics** | **Reference (paper)** | **Cohort** | **Sample size** | **GWAS summary statistics link** |
| --- | --- | --- | --- | --- |
| RVESV | Aung N et al.^36^ | UK Biobank | 29,506 | [(1)](https://www.ebi.ac.uk/gwas/publications/35697868) |
| RVEDV |  |  |  |  |
| RVEF |  |  |  |  |
| LVM | Aung N et al.^37^ |  | 16,923 | [GWAS Catalog (ebi.ac.uk)](https://www.ebi.ac.uk/gwas/publications/31554410) |
| LVSV | Pirruccello JP et al.^38^ |  | 40,000 | [Downloads \| Cardiovascular Disease Knowledge Portal (hugeamp.org)](https://cvd.hugeamp.org/downloads.html) |
| LVEDV |  |  |  |  |
| LVESV |  |  |  |  |
| Native Myocardial T1 | Nauffal V et al.^39^ |  | 41,505 | [Downloads \| Cardiovascular Disease Knowledge Portal (hugeamp.org)](https://cvd.hugeamp.org/downloads.html) |
| LVEF | Pirruccello JP.^38^ |  | 40,000 | [Downloads \| Cardiovascular Disease Knowledge Portal (hugeamp.org)](https://cvd.hugeamp.org/downloads.html) |
| ASI | Fung K.^40^ |  | 127,121 | [GWAS Catalog (ebi.ac.uk)](https://www.ebi.ac.uk/gwas/publications/31235810) |
| AoD | Francis C.^41^ |  | 32,590 | [GENOME-WIDE ASSOCIATIONS OF AORTIC DISTENSIBILITY SUGGEST CAUSALITY FOR AORTIC ANEURYSMS AND BRAIN WHITE MATTER HYPERINTENSITIES \| Imperial College London](https://data.hpc.imperial.ac.uk/resolve/?doi=10653) |

**Footnote Table 6:** Studies included in the Mendelian randomisation analysis. AoD – aortic distensibility; ASI – arterial stiffness index; LV – left ventricle; LVEDV – LV end-diastolic volume; LVEF – LV ejection fraction; LVESV – LV end-systolic volume; LVM – LV mass; LVSV – LV stroke volume; RV – right ventricle; RVEDV – RV end-diastolic volume; RVEF – RV ejection fraction; RVESV – RV end-systolic volume.

# Table 7: Characteristics of genetic variants as instrumental variables (IVs)

| **RsID** | **Chr** | **BP g** **38** | **EA** | **NEA** | **Beta** | **SE** | **P value** |
| --- | --- | --- | --- | --- | --- | --- | --- |
| **rs12117799**  (rs112438759, R^2^ = 0.18) | 1 | 113775982 | T | C | -0.146 | 0.018 | 1.19E-16 |
| rs2476601 | 1 | 113834946 | G | A | -0.544 | 0.020 | 3.75E-168 |
| rs12137270 | 1 | 116721714 | T | C | 0.085 | 0.015 | 2.20E-08 |
| rs11810143 | 1 | 161510859 | G | A | 0.115 | 0.020 | 3.63E-09 |
| rs4916340 | 1 | 173378335 | T | G | 0.084 | 0.015 | 1.52E-08 |
| rs2240336 | 1 | 17347907 | T | C | -0.086 | 0.013 | 1.89E-10 |
| rs6429207 | 1 | 235635034 | C | A | -0.101 | 0.017 | 2.76E-09 |
| **rs745368**  (rs867435, R^2^ =0.98) | 1 | 2592766 | T | C | -0.089 | 0.014 | 5.92E-11 |
| rs2045793 | 1 | 38169790 | G | A | -0.097 | 0.015 | 2.37E-10 |
| rs6659932 | 1 | 67336688 | C | A | -0.102 | 0.018 | 7.19E-09 |
| rs12474386 | 2 | 100240555 | A | G | -0.100 | 0.013 | 1.03E-14 |
| rs4853458 | 2 | 191094763 | G | A | -0.124 | 0.015 | 9.84E-17 |
| rs3087243 | 2 | 203874196 | A | G | -0.121 | 0.013 | 2.72E-21 |
| rs10173253 | 2 | 30220594 | A | G | 0.078 | 0.014 | 1.32E-08 |
| rs34695944 | 2 | 60897715 | C | T | 0.104 | 0.013 | 1.03E-15 |
| rs17534670 | 2 | 65388294 | A | G | -0.090 | 0.013 | 9.80E-13 |
| **rs4472039**  (rs5019428, R^2^: 0.99) | 3 | 17005631 | C | T | 0.092 | 0.014 | 7.69E-12 |
| rs9310852 | 3 | 27743506 | G | A | 0.079 | 0.013 | 1.08E-09 |
| rs12506688 | 4 | 26102491 | T | C | 0.134 | 0.014 | 3.71E-22 |
| rs2664035 | 4 | 48218822 | A | G | 0.082 | 0.013 | 3.85E-10 |
| rs2561477 | 5 | 103273223 | A | G | -0.092 | 0.014 | 4.96E-11 |
| rs5745271 | 5 | 10690637 | G | T | 0.075 | 0.013 | 1.14E-08 |
| rs7731626 | 5 | 56148856 | A | G | -0.186 | 0.016 | 4.20E-33 |
| rs548234 | 6 | 106120159 | T | C | -0.077 | 0.013 | 7.30E-09 |
| rs17264332 | 6 | 137684378 | G | A | 0.168 | 0.015 | 4.14E-29 |
| rs6570194 | 6 | 137921501 | C | A | 0.137 | 0.024 | 5.53E-09 |
| rs113532504 | 6 | 15195451 | T | C | 0.122 | 0.022 | 1.61E-08 |
| rs2451279 | 6 | 159094045 | G | A | 0.086 | 0.013 | 1.84E-10 |
| **rs3093024**  (rs3093017, R^2^: 0.98) | 6 | 167119305 | G | A | -0.111 | 0.013 | 3.14E-18 |
| rs3130396 | 6 | 30255713 | C | T | 0.206 | 0.013 | 5.81E-58 |
| **rs9268951**  rs9268949, R^2^ = 0.95) | 6 | 32466130 | A | G | 0.577 | 0.014 | 0 |
| rs213196 | 6 | 33229767 | T | C | -0.235 | 0.025 | 1.98E-21 |
| rs72928038 | 6 | 90267049 | A | G | 0.107 | 0.018 | 6.28E-09 |
| **rs3807307**  rs3778754, R^2^ = 0.98) | 7 | 128939148 | C | T | 0.108 | 0.013 | 1.11E-16 |
| rs678347 | 8 | 101451374 | A | G | -0.084 | 0.014 | 3.25E-09 |
| rs2409780 | 8 | 11480078 | C | T | 0.108 | 0.016 | 2.23E-11 |
| rs16903108 | 8 | 128564231 | C | T | -0.142 | 0.021 | 1.96E-11 |
| rs998731 | 8 | 80183160 | T | C | 0.077 | 0.014 | 2.00E-08 |
| rs10985070 | 9 | 120873843 | A | C | -0.079 | 0.013 | 1.92E-09 |
| rs10972201 | 9 | 34707376 | A | G | 0.099 | 0.014 | 4.28E-13 |
| rs2793108 | 10 | 31090176 | T | C | 0.070 | 0.013 | 4.62E-08 |
| rs706778 | 10 | 6056986 | T | C | 0.105 | 0.013 | 3.37E-16 |
| rs77509998 | 10 | 62054031 | T | G | 0.127 | 0.017 | 6.84E-14 |
| rs10905284 | 10 | 8073399 | A | C | -0.098 | 0.015 | 3.81E-11 |
| **rs7125333**  (rs4938573, R^2^ = 0.99) | 11 | 118872015 | C | T | 0.123 | 0.017 | 1.41E-13 |
| rs3184504 | 12 | 111446804 | C | T | -0.087 | 0.014 | 1.04E-10 |
| **rs238516**  rs1696466, R^2^ = 1) | 12 | 57723572 | C | T | 0.071 | 0.013 | 4.23E-08 |
| rs9603608 | 13 | 39744682 | C | A | -0.109 | 0.014 | 2.32E-15 |
| rs7170151 | 15 | 38554477 | T | C | 0.102 | 0.014 | 9.49E-13 |
| rs8026898 | 15 | 69699078 | A | G | 0.138 | 0.014 | 9.90E-23 |
| rs115284761 | 15 | 77034495 | C | T | -0.117 | 0.021 | 2.78E-08 |
| **rs933573**  (rs4584833, R^2^ = 0.85) | 16 | 11699539 | T | C | 0.072 | 0.013 | 4.48E-08 |
| **rs4795398**  (rs62067029, R^2^ = 0.91) | 17 | 39881926 | T | C | 0.077 | 0.013 | 1.13E-09 |
| **rs8087237**  (rs641085, R^2^ = 0.99) | 18 | 12834360 | A | C | -0.087 | 0.013 | 1.08E-11 |
| rs4239702 | 20 | 46120612 | C | T | 0.104 | 0.014 | 5.98E-13 |
| rs8133843 | 21 | 35365944 | A | G | 0.085 | 0.014 | 1.33E-09 |
| rs11089637 | 22 | 21624807 | C | T | 0.111 | 0.018 | 2.12E-10 |
| rs137687 | 22 | 39344073 | A | G | -0.089 | 0.013 | 7.79E-12 |

**Footnote Table 7:** Variants shown in bold are proxies for the original variants listed in brackets. RsID - variants ID; Chr - Chromosome; BP - base pair location; EA - effect allele; NEA - non-effect allele; SE - standard error.

# Table 8: Summary of GWAS for cardiovascular conditions in East Asian populations

| **Outcome** | **Reference (paper)** | **Cohort** | **Cases** | **Control** | **Sample size** | **GWAS summary statistics link** |
| --- | --- | --- | --- | --- | --- | --- |
| Ischaemic heart  disease | Kanai M et al. ^1^ | BioBank Japan | 32512 | 146214 | 178726 | <https://pheweb.jp/pheno/CAD> |
| Acute myocardial  infarction | Sakaue S et al. ^2^ |  | 14992 | 146214 | 161206 | <https://pheweb.jp/pheno/MI> |
| Heart failure |  |  | 10540 | 168186 | 178726 | <https://pheweb.jp/pheno/CHF> |
| Atrial fibrillation |  |  | 4150 | 155540 | 159690 | <https://pheweb.jp/pheno/Atrial_Flutter> |
| Dilated cardiomyopathy |  |  | 417 | 177745 | 178162 | <https://pheweb.jp/pheno/Dilated_Cardiomyopathy> |
| Hypertrophic  cardiomyopathy |  |  | 383 | 177745 | 178128 | <https://pheweb.jp/pheno/Hypertrophic_Cardiomyopathy> |
| Stroke |  |  | 22664 | 152022 | 174686 | <https://pheweb.jp/pheno/IS> |
| Peripheral arterial  disease |  |  | 4112 | 173601 | 177713 | <https://pheweb.jp/pheno/PAD> |

**Footnote Table 8:** This table summarises the genome-wide association studies (GWAS) used to define cardiovascular outcomes in the Mendelian randomisation analysis conducted in the East Asian population. For each outcome, the table provides the study cohort, number of cases and controls, total sample size, and a link to the publicly available summary statistics, where available. All datasets reflect East Asian ancestry populations. GWAS - genome wide association studies.

^1^ Kanai M, Ulirsch JC, Karjalainen J, Kurki M, Karczewski KJ, Fauman E, et al. Insights from complex trait fine-mapping across diverse populations. medRxiv [Internet]. 2021; Available from: <https://www.medrxiv.org/content/early/2021/09/05/2021.09.03.21262975>.

^2^ Sakaue S, Kanai M, Tanigawa Y, Karjalainen J, Kurki M, Koshiba S, et al. A cross-population atlas of genetic associations for 220 human phenotypes. Nat Genet. 2021 Oct;53(10):1415–24.

# Table 9: Power calculation for Mendelian Randomisation analysis of RA and CVD

| **Exposure** | **Explained variance** | **Cardiac outcome** | **Cases** | **Control** | **Sample size** | **Proportion of cases** | **Alpha** | **Power** | **OR** |
| --- | --- | --- | --- | --- | --- | --- | --- | --- | --- |
| Rheumatoid  arthritis | 0.14 | Ischaemic heart disease | 69008 | 343173 | 412181 | 0.167421594 | 0.0056 | 0.9 | 1.0456 |
|  |  | Acute myocardial infarction | 26060 | 343079 | 369139 | 0.070596713 |  |  | 1.07 |
|  |  | Heart failure | 29672 | 382509 | 412181 | 0.071987792 |  |  | 1.066 |
|  |  | Atrial fibrillation | 50743 | 210652 | 261395 | 0.194123836 |  |  | 1.054 |
|  |  | Any arrhythmia | 74000 | 239778 | 313778 | 0.235835527 |  |  | 1.0463 |
|  |  | Non-ischaemic cardiomyopathy | 1,754 | 340,815 | 342569 | 0.005120136 |  |  | 1.26 |
|  |  | Stroke | 43,132 | 297,867 | 340999 | 0.126487174 |  |  | 1.056 |
|  |  | Peripheral vascular disease | 2,489 | 381,977 | 384466 | 0.006473914 |  |  | 1.217 |
|  |  | Venous thromboembolism | 21,021 | 391,160 | 412181 | 0.05099944 |  |  | 1.077 |

**Footnote Table 9:** This table presents the power calculations for detecting associations between RA and various cardiac outcomes using MR analysis. The table includes the explained variance of the exposure, the number of cases and controls, the sample size, the proportion of cases in the sample, the alpha level, power of the study and the OR detectable with 90% power for each cardiac outcome. OR - odds ratio; CVD - cardiovascular disease.

# Table 10: Non-MICE Model for the Association of Rheumatoid Arthritis with Prevalent Cardiovascular Diseases and Vascular Risk Factors

| **Outcome** | **Model 2 (non-MICE)** |
| --- | --- |
|  | OR (95% CI)  p-value |
| Ischaemic Heart Disease | 1.43 (1.07,1.91)  0.02 |
| Acute Myocardial Infarction | 1.85 (1.28,2.66)  0.001 |
| Heart Failure | 1.99 (1.04,3.80)  0.04 |
| Atrial Fibrillation | 2.49(1.45,4.29)  0.001 |
| Any Arrhythmia | 1.41 (0.84,2.38)  0.19 |
| Non-Ischaemic Cardiomyopathy | 2.65 (0.84,8.38)  0.18 |
| Pericardial Disease | 4.71 (2.07,10.74)  <0.001 |
| Stroke | 1.01 (0.62,1.64)  0.97 |
| Peripheral Vascular Disease | 1.85 (0.90,3.81)  0.10 |
| Venous Thromboembolism | 1.88 (1.28,2.76)  0.001 |
| Diabetes | 1.05 (0.80,1.39)  0.72 |
| Hypertension | 1.49 (1.26,1.77)  <0.001 |
| Hypercholesterolaemia | 1.10 (0.90,1.34)  0.37 |

**Footnote Table 10:** Model 2 is adjusted for age, sex, physical activity, alcohol consumption, current smoking, educational level, deprivation, diabetes, hypertension, hypercholesterolaemia, total cholesterol/HDL ratio and CRP. N=269,845

CI – Confidence Interval, OR – Odds Ratio, MICE - multiple imputation by chained equations

# Table 11: Non-MICE Model for the Association of Rheumatoid Arthritis with Incident Cardiovascular Diseases

| **Outcome** | **Model 2 (non-MICE)** |
| --- | --- |
|  | HR (95% CI)  p-value |
| Ischaemic Heart Disease | 1.44 (1.18,1.77)  <0.001 |
| Acute Myocardial Infarction | 1.51 (1.05,2.18)  0.03 |
| Heart Failure | 1.55 (1.20,2.00)  0.001 |
| Atrial Fibrillation | 1.17 (0.92,1.49)  0.21 |
| Any Arrhythmia | 1.18 (0.91,1.52)  0.21 |
| Non-Ischaemic Cardiomyopathy | 1.21 (0.57,2.54)  0.62 |
| Pericardial Disease | 2.20 (1.27,3.80)  <0.001 |
| Stroke | 1.10 (0.73,1.66)  0.65 |
| Peripheral Vascular Disease | 1.60 (1.10,2.33)  0.01 |
| Venous Thromboembolism | 1.13 (0.72,1.77)  0.61 |

**Footnote Table 11:** Model 2 is adjusted for age, sex, physical activity, alcohol consumption, current smoking, educational level, deprivation, diabetes, hypertension, hypercholesterolaemia, total cholesterol/HDL ratio and CRP. Estimates from fully adjusted model. N=269,845.

CI – Confidence Interval, HR – Hazard Ratio, MICE - multiple imputation by chained equations

# Table 12: MR estimates of rheumatoid arthritis associated with the risk of cardiovascular diseases

|  | **AMI** | **AA** | **AF** | **HF** | **IHD** | **NIHD** | **PVD** | **Stroke** | **VTE** |
| --- | --- | --- | --- | --- | --- | --- | --- | --- | --- |
| IVW beta | 0.049 | 0.0408 | 0.036 | 0.037 | 0.027 | 0.018 | 0.004 | 0.019 | 0.017 |
| IVW SE | 0.015 | 0.009 | 0.014 | 0.013 | 0.009 | 0.041 | 0.037 | 0.009 | 0.011 |
| IVW (P) | 0.001 | 0.00008 | 0.013 | 0.006 | 0.004 | 0.646 | 0.906 | 0.055 | 0.138 |
| IVW (CP) | **0.009** | **0.0007** | 0.117 | 0.054 | **0.036** | 1 | 1 | 0.495 | 1 |
| MR Egger (P) | 0.005 | 0.004 | 0.086 | 0.001 | 0.009 | 0.654 | 0.745 | 0.657 | <0.001 |
| W median (P) | <0.001 | <0.001 | <0.001 | <0.001 | <0.001 | 0.586 | 0.860 | 0.762 | 0.389 |
| W mode (P) | <0.001 | <0.001 | <0.001 | <0.001 | <0.001 | 0.503 | 0.403 | 0.320 | 0.025 |
| Egger_intercept (P) | 0.168 | 0.283 | 0.433 | 0.029 | 0.196 | 0.820 | 0.631 | 0.419 | <0.001 |
| MR_PRESSO_GT(P) | <0.001 | <0.001 | <0.001 | 0.001 | <0.001 | 0.084 | 0.016 | <0.001 | 0.006 |
| MR_PRESSO_DT(P) | 0.867 | 0.768 | 0.82 | NA | 0.46 | NA | NA | 0.59 | 0.219 |

**Footnote Table 12:** Results from the main analysis using the IVW method and complementary analyses. Significant results in the main analysis, corrected p-value and complementary analyses are highlighted in bold. IVW - Inverse variance weighted; SE - standard error; P - p-value; CP - corrected p-value using Bonferroni method; W - weighted; GT - global test; DT - distortion test; AMI - acute myocardial infarction; AA - any arrhythmia; AF - atrial fibrillation; HF - heart failure; IHD - ischemic heart disease; NIHD - non-ischaemic cardiomyopathy; PVD - peripheral vascular disease; VT - venous thromboembolism.

# Table 13: Characteristics of genetic variants used as instrumental variables (IVs) in the East Asian Mendelian Randomisation analysis

| **RsID** | **Chr** | **Position**  **(GRCh37)** | **EA** | **OA** | **Beta** | **SE** | **P-value** |
| --- | --- | --- | --- | --- | --- | --- | --- |
| rs2240335 | 1 | 17674537 | A | C | -0.1786 | 0.0167 | 1.01E-26 |
| rs2235920 | 1 | 17420398 | G | C | 0.1021 | 0.0168 | 1.25E-09 |
| rs60389769 | 1 | 2717470 | A | G | -0.1072 | 0.0177 | 1.41E-09 |
| rs9729284 | 1 | 38373989 | T | C | 0.106 | 0.0184 | 7.75E-09 |
| rs11889341 | 2 | 1.92E+08 | T | C | 0.1284 | 0.0176 | 2.68E-13 |
| rs231836 | 2 | 2.05E+08 | T | C | -0.105 | 0.0168 | 3.92E-10 |
| rs11891046 | 2 | 1.01E+08 | G | C | -0.1043 | 0.0167 | 3.94E-10 |
| rs6546883 | 2 | 74217856 | G | A | -0.1323 | 0.023 | 8.73E-09 |
| rs58107865 | 4 | 1.09E+08 | C | G | -0.1736 | 0.023 | 4.22E-14 |
| rs56817615 | 4 | 10718846 | G | A | 0.0926 | 0.0167 | 3.06E-08 |
| rs7731626 | 5 | 55444683 | A | G | -0.2288 | 0.0327 | 2.74E-12 |
| rs147533526 | 5 | 40563206 | A | C | -0.1582 | 0.0282 | 2.01E-08 |
| rs116920857 | 6 | 32390381 | C | A | 0.7526 | 0.024 | 1.00E-200 |
| rs17204294 | 6 | 32492179 | A | T | -0.5707 | 0.0252 | 1.13E-113 |
| rs34551973 | 6 | 32547106 | C | T | 0.614 | 0.0358 | 4.10E-66 |
| rs3873453 | 6 | 32683382 | T | C | -0.3685 | 0.0248 | 6.54E-50 |
| rs3093018 | 6 | 1.68E+08 | T | C | -0.2056 | 0.0173 | 1.36E-32 |
| rs2233424 | 6 | 44233921 | T | C | 0.1843 | 0.0198 | 1.57E-20 |
| rs9268652 | 6 | 32409056 | G | A | 0.354 | 0.039 | 1.18E-19 |
| rs115155836 | 6 | 31319416 | G | C | 0.5426 | 0.0702 | 1.11E-14 |
| rs6570194 | 6 | 1.38E+08 | C | A | 0.1882 | 0.031 | 1.33E-09 |
| rs78019882 | 6 | 14111387 | G | C | 0.1294 | 0.0221 | 4.71E-09 |
| rs118160129 | 6 | 30967305 | A | G | -0.3385 | 0.059 | 9.46E-09 |
| rs112289675 | 6 | 33178668 | C | T | -0.4539 | 0.0823 | 3.45E-08 |
| rs3757387 | 7 | 1.29E+08 | C | T | 0.1767 | 0.0257 | 6.03E-12 |
| rs34130487 | 7 | 99759205 | T | C | -0.1128 | 0.0168 | 1.80E-11 |
| rs2618476 | 8 | 11352541 | C | T | 0.1445 | 0.0187 | 1.04E-14 |
| rs7902146 | 10 | 63801030 | T | C | -0.1268 | 0.017 | 9.88E-14 |
| rs3956896 | 10 | 64066141 | G | A | -0.1934 | 0.027 | 7.68E-13 |
| rs7097397 | 10 | 50025396 | A | G | -0.1134 | 0.0177 | 1.34E-10 |
| rs643610 | 10 | 6521147 | A | G | 0.1171 | 0.0183 | 1.46E-10 |
| rs11235575 | 11 | 72419549 | T | A | -0.1167 | 0.0183 | 1.70E-10 |
| rs510372 | 11 | 64115137 | T | C | -0.1061 | 0.0194 | 4.75E-08 |
| rs2819464 | 14 | 1.05E+08 | G | T | 0.1812 | 0.0202 | 2.86E-19 |
| rs3783795 | 14 | 61925334 | A | G | 0.1098 | 0.0195 | 1.93E-08 |
| rs8035957 | 15 | 38838264 | C | T | 0.1029 | 0.0169 | 1.08E-09 |
| rs9933582 | 16 | 86016026 | G | T | 0.1063 | 0.0176 | 1.41E-09 |
| rs657555 | 18 | 12847136 | T | C | -0.109 | 0.017 | 1.44E-10 |
| rs17207042 | 18 | 67537351 | C | T | -0.0951 | 0.0166 | 1.05E-08 |
| rs55882956 | 19 | 10469919 | A | G | -0.4307 | 0.0729 | 3.46E-09 |
| rs1883832 | 20 | 44746982 | C | T | 0.1052 | 0.0172 | 8.65E-10 |
| rs2075876 | 21 | 45709153 | A | G | 0.1329 | 0.0183 | 3.56E-13 |
| rs1807560 | 22 | 39748912 | A | G | 0.1846 | 0.025 | 1.47E-13 |

**Footnote Table 13:** Variants used as instrumental variables in the East Asian Mendelian Randomisation analysis. RsID - variants ID; Chr – chromosome; EA – effect allele; OA – other allele; SE – standard error; P-value – association between RsID and rheumatoid arthritis in the East Asian GWAS. Position based on GRCh37 genome build.

# Table 14: MR estimates of rheumatoid arthritis associated with the risk of cardiovascular diseases in an East Asian population

|  | **AMI** | **AF** | **DC** | **HC** | **HF** | **IHD** | **PAD** | **Stroke** |
| --- | --- | --- | --- | --- | --- | --- | --- | --- |
| IVW beta | 0.043 | -0.015 | 0.106 | 0.064 | 0.037 | 0.041 | 0.017 | 0.015 |
| IVW SE | 0.027 | 0.026 | 0.081 | 0.092 | 0.016 | 0.018 | 0.027 | 0.012 |
| IVW (P) | 0.113 | 0.559 | 0.191 | 0.481 | **0.023** | **0.027** | 0.520 | 0.202 |
| IVW (CP) | 0.904 | 1 | 1 | 1 | 0.184 | 0.216 | 1 | 1 |
| MR Egger (P) | 0.008 | 0.697 | 0.292 | 0.939 | 0.026 | 0.027 | 0.425 | 0.413 |
| W median (P) | 0.0001 | 0.719 | 0.135 | 0.543 | 0.053 | 0.005 | 0.478 | 0.964 |
| W mode (P) | 0.005 | 0.680 | 0.237 | 0.494 | 0.036 | 0.022 | 0.437 | 0.950 |
| Egger_intercept (P) | **0.031** | 0.933 | 0.678 | 0.707 | 0.215 | 0.206 | 0.589 | 0.891 |
| MR_PRESSO_GT(P) | <0.001 | 0.437 | 0.380 | 0.170 | 0.617 | <0.001 | 0.337 | 0.683 |
| MR_PRESSO_DT(P) | **0.004** |  |  |  |  | 0.572 |  |  |

**Footnote Table 14:** Results from the main analysis using the inverse IVW method and complementary approaches (MR Egger, weighted median, weighted mode, MR-PRESSO). Significant results (p < 0.05) and Bonferroni-corrected p-values (CP < 0.00625) are shown in bold. Egger intercept p-values assess directional pleiotropy; MR-PRESSO global test (GT) and distortion test (DT) evaluate horizontal pleiotropy. Abbreviations: IVW – inverse variance weighted; SE – standard error; CP – Bonferroni-corrected p-value; GT – global test; DT – distortion test; W – weighted; AF – atrial fibrillation; HF – heart failure; IHD – ischaemic heart disease; MI – myocardial infarction; DC – dilated cardiomyopathy; HC – hypertrophic cardiomyopathy; PAD – peripheral artery disease; Stroke – any stroke subtype.

# Table 15: Baseline Cardiac Magnetic Resonance Metrics Comparison Between Participants Without Rheumatoid Arthritis (No RA) and With Rheumatoid Arthritis (RA)

| **CMR metrics** | **No RA** | **RA** |
| --- | --- | --- |
| LAVmax(i) (mL/m^2^) | 72.92 (23.24) | 76.24 (21.24) |
| LAEF (%) | 61.26 (9.15) | 61.96 (6.34) |
| LV WT (mm) | 5.69 (0.77) | 5.82 (0.83) |
| LVM(i) (g/m^2^) | 86.23 (22.24) | 87.70 (20.45) |
| LVEDV(i) (mL/m^2^) | 148.40 (33.83) | 148.04 (26.92) |
| LVM : LVEDV (g/mL) | 0.58 (0.09) | 0.59 (0.09) |
| LVSV(i) (mL/m^2^) | 87.79 (19.24) | 87.83 (16.79) |
| LVEF (%) | 59.56 (6.06) | 59.35 (4.54) |
| RAVmax(i) (mL/m^2^) | 86.15 (27.44) | 89.23 (27.27) |
| RAEF (%) | 47.11 (9.17) | 49.04 (7.53) |
| RVEDV(i) (mL/m^2^) | 156.85 (37.34) | 158.97 (39.38) |
| RVSV(i) (mL/m^2^) | 89.23 (20.38) | 89.38 (22.49) |
| RVEF (%) | 57.33 (6.14) | 56.51 (6.24) |
| AoD (10^-3^ mmHg^-1^) | 1.31 (0.69) | 1.88 (1.37) |

**Footnote Table 15:** Results are presented as means with standard deviations. The "i" denotes values indexed to body surface area (m^2^), calculated using the Mosteller formula.

AoD - Aortic distensibility, LAEF - Left atrial ejection fraction, LAVmax(i) - Left atrial maximum volume indexed, LVEF - Left ventricular ejection fraction, LV WT - Left ventricular wall thickness, LVEDV(i) - Left ventricular end-diastolic volume indexed, LVM : LVEDV - Ratio of left ventricular mass to left ventricular end-diastolic volume, LVM(i) - Left ventricular mass indexed, LVSV(i) - Left ventricular stroke volume indexed, RA - Rheumatoid Arthritis, RAEF - Right atrial ejection fraction, RAVmax(i) - Right atrial maximum volume indexed, RVEF - Right ventricular ejection fraction, RVEDV(i) - Right ventricular end-diastolic volume indexed, RVSV(i) - Right ventricular stroke volume indexed.

# Table 16: Association of Rheumatoid Arthritis with Cardiac Magnetic Resonance Metrics

| **CMR metrics** | **Model 1** | **Model 2** | **Model 3** |
| --- | --- | --- | --- |
|  | β (95% CI)  p-value | | |
| LAVmax(i) (mL/m^2^) | 0.17 (-0.25,0.60)  0.42 | 0.24 (-0.23,0.70)  0.32 | 0.19 (-0.27,0.65)  0.42 |
| LAEF (%) | 0.10 (-0.33,0.54)  0.65 | 0.14 (-0.32,0.61)  0.55 | 0.17 (-0.30,0.64)  0.48 |
| LV WT (mm) | 0.16 (-0.17,0.48)  0.34 | 0.11 (-0.24,0.47)  0.53 | 0.02 (-0.33,0.36)  0.93 |
| LVM(i) (g/m^2^) | 0.09 (-0.20,0.39)  0.54 | 0.12 (-0.20,0.44)  0.48 | 0.05 (-0.27,0.36)  0.78 |
| LVEDV(i) (mL/m^2^) | 0.04 (-0.30,0.38)  0.83 | 0.10 (-0.26,0.47)  0.58 | 0.09 (-0.28,0.45)  0.64 |
| LVM : LVEDV (g/mL) | 0.08 (-0.32,0.47)  0.70 | 0.003 (-0.41,0.42)  0.99 | -0.09 (-0.51,0.32)  0.65 |
| LVSV(i) (mL/m^2^) | 0.05 (-0.32,0.42)  0.80 | 0.17 (-0.23,0.57)  0.41 | 0.16 (-0.24,0.55)  0.44 |
| LVEF (%) | -0.05 (-0.46,0.36)  0.82 | 0.06 (-0.38,0.50)  0.79 | 0.07 (-0.38,0.51)  0.77 |
| RAVmax(i) (mL/m^2^) | 0.14 (-0.26,0.54)  0.48 | 0.22 (-0.22,0.65)  0.33 | 0.23 (-0.20,0.67)  0.29 |
| RAEF (%) | 0.20 (-0.23,0.62)  0.36 | 0.10 (-0.35,0.56)  0.66 | 0.09 (-0.37,0.55)  0.70 |
| RVEDV(i) (mL/m^2^) | 0.11 (-0.21,0.43)  0.51 | 0.24 (-0.11,0.59)  0.17 | 0.24 (-0.10,0.59)  0.17 |
| RVSV(i) (mL/m^2^) | 0.05 (-0.31,0.42)  0.78 | 0.21 (-0.18,0.60)  0.29 | 0.21 (-0.18,0.60)  0.30 |
| RVEF (%) | -0.15 (-0.55,0.25)  0.46 | -0.12 (-0.55,0.32)  0.60 | -0.12 (-0.56,0.31)  0.58 |
| AoD (10^-3^ mmHg^-1^) | -0.38 (-0.77,0.008)  0.06 | -0.37 (-0.79,0.06)  0.09 | -0.34 (-0.76,0.09)  0.12 |

**Footnote Table 16:** Results from linear regression models evaluating the association between rheumatoid arthritis (RA) and cardiac magnetic resonance (CMR) metrics. The results are reported as standardized beta coefficients (β), 95% confidence intervals (CI), and p-values. Three models were used: Model 1 is adjusted for age and sex; Model 2 includes additional adjustments for ethnicity, physical activity, alcohol consumption, current smoking, educational level, and deprivation; Model 3 further adjusts for diabetes, hypertension, and hypercholesterolaemia.

AoD - Aortic distensibility, LAEF - Left atrial ejection fraction, LAVmax(i) - Left atrial maximum volume indexed, LVEF - Left ventricular ejection fraction, LV WT - Left ventricular wall thickness, LVEDV(i) - Left ventricular end-diastolic volume indexed, LVM : LVEDV - Ratio of left ventricular mass to left ventricular end-diastolic volume, LVM(i) - Left ventricular mass indexed, LVSV(i) - Left ventricular stroke volume indexed, RAEF - Right atrial ejection fraction, RAVmax(i) - Right atrial maximum volume indexed, RVEF - Right ventricular ejection fraction, RVEDV(i) - Right ventricular end-diastolic volume indexed, RVSV(i) - Right ventricular stroke volume indexed.

# Table 17: MR estimates of rheumatoid arthritis and CMR metrics

|  | **RVESV** | **RVEDV** | **RVEF** | **LVM** | **LVSV** | **LVEDV** | **LVESV** | **T1** | **LVEF** | **ASI** | **AOD** |
| --- | --- | --- | --- | --- | --- | --- | --- | --- | --- | --- | --- |
| IVW beta | -0.033 | -0.025 | 0.029 | -0.029 | 0.13 | 0.009 | 0.122 | 0.079 | 0.724 | 0.213 | 0.341 |
| IVW SE | 0.011 | 0.011 | 0.010 | 0.012 | 0.009 | 0.009 | 0.008 | 0.007 | 0.007 | 0.005 | 0.007 |
| IVW (P) | 0.00004 | 0.00100 | 0.00259 | 0.01398 | 0.02400 | 0.04200 | 0.04800 | 0.05489 | 0.23575 | 0.59563 | 0.74153 |
| IVW (CP) | **0.0004** | **0.005** | **0.0094** | **0.0385** | 0.0528 | 0.0754 | 0.0754 | 0.0754 | 0.2884 | 0.655 | 0.742 |
| MR Egger (P) | 0.218 | 0.181 | 0.509 | 0.580 | 0.196 | 0.258 | 0.459 | 0.233 | 0.735 | 0.656 | 0.117 |
| W median (P) | 0.056 | 0.084 | 0.372 | 0.097 | 0.013 | 0.009 | 0.122 | 0.079 | 0.724 | 0.213 | 0.341 |
| W mode (P) | 0.016 | 0.020 | 0.168 | 0.161 | 0.020 | 0.019 | 0.231 | 0.163 | 0.867 | 0.694 | 0.284 |
| Egger_intercept (P) | 0.367 | 0.739 | 0.189 | 0.295 | 0.457 | 0.747 | 0.723 | 0.904 | 0.213 | 0.860 | 0.092 |
| MR_PRESSO_GT(P) | <0.01 | <0.01 | 0.05 | 0.15 | <0.01 | <0.01 | <0.01 | 0.94 | 0.02 | 0.02 | 0.19 |
| MR_PRESSO_DT(P) | 0.5 | 0.33 |  |  | 0.55 | 0.76 | 0.22 |  |  | 0.33 |  |

**Footnote Table 17:** Results from the main analysis using the IVW method and complementary analyses. The significant results in the main analysis and corrected p-value, but not in the complementary analyses, are highlighted in bold. IVW - Inverse variance weighted; SE - standard error; P - p-value; CP - corrected p-value using Bonferroni method; W - weighted; GT - global test; DT - distortion test; RVESV - RV end-systolic volume; RVEDV - RV end-diastolic volume; RVEF - RV ejection fraction; LVM - LV mass; LVSV - LV stroke volume; LVEDV - LV end-diastolic volume; LVESV - LV end-systolic volume; LVEF - LV ejection fraction; ASI - arterial stiffness index; AoD - aortic distensibility.
