# Supplementary material for: Rheumatoid arthritis and cardiovascular disease associations in the UK Biobank
Source: BMC Med. 2025 Nov 3;23:605. doi: 10.1186/s12916-025-04431-1 (PMC12581239; doi:10.1186/s12916-025-04431-1)
Supplement: Supplementary file 2 — Additional file 2. Figure 1: Associations between RA and CMR metrics [file 12916_2025_4431_MOESM2_ESM.docx]

Additional file 2

# **Figure 1: Associations between RA and CMR metrics**

**
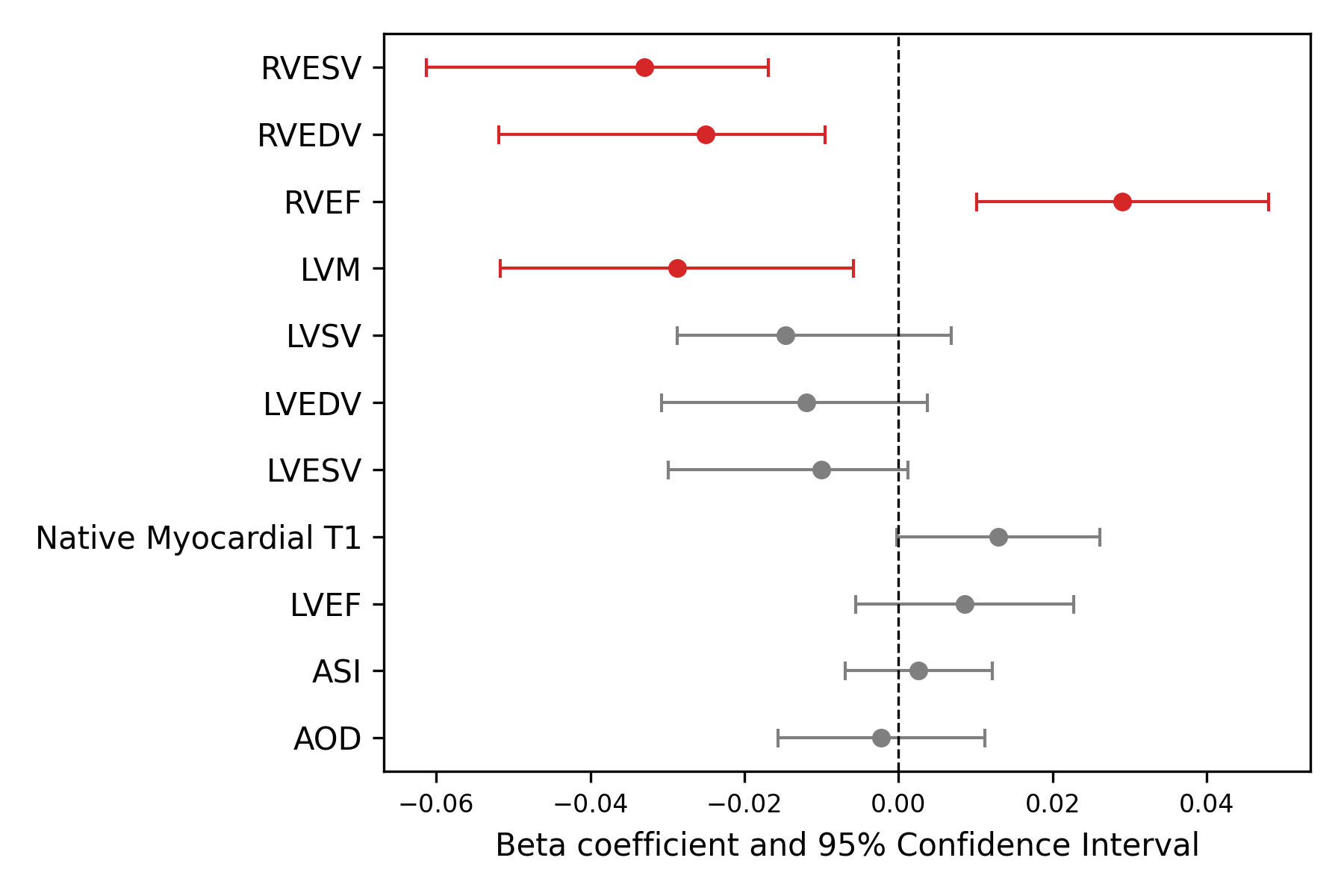
**

**Footnote Figure 1:** Forest plot of Mendelian Randomisation estimates of RA associations with CMR metrics. Point estimates reflect odds ratios (OR) with 95% confidence intervals presented as horizontal line segments. The x-axis represents the odds ratio (OR) along with the 95% confidence interval (CI). The y-axis lists the CMR metrics analysed: RVESV - RV end-systolic volume; RVEDV - RV end-diastolic volume; RVEF - RV ejection fraction; LVM - LV mass; LVSV - LV stroke volume; LVEDV - LV end-diastolic volume; LVESV - LV end-systolic volume; LVEF - LV ejection fraction; ASI - arterial stiffness index; AoD - aortic distensibility.
